# TCA Cycle Replenishing Pathways in Photosynthetic Purple Non-Sulfur Bacteria Growing with Acetate

**DOI:** 10.3390/life11070711

**Published:** 2021-07-19

**Authors:** Ekaterina Petushkova, Ekaterina Mayorova, Anatoly Tsygankov

**Affiliations:** 1 Pushchino Scientific Center for Biological Research, Institute of Basic Biological Problems Russian Academy of Sciences, 2, Institutskaya Str, 142290 Pushchino, Moscow Region, Russia; peteka2008@gmail.com (E.P.); ekaterina.majorova.97@mail.ru (E.M.); 2 Pushchino State Institute of Natural Science, The Federal State Budget Educational Institution of Higher Education, 3, Prospekt Nauki, 142290 Pushchino, Moscow Region, Russia

**Keywords:** purple non-sulfur bacteria, tricarboxylic acid cycle, anaplerotic pathways, glyoxylate pathway, ethylmalonyl-CoA pathway, citramalate cycle, methylaspartate cycle

## Abstract

Purple non-sulfur bacteria (PNSB) are anoxygenic photosynthetic bacteria harnessing simple organic acids as electron donors. PNSB produce *a*-aminolevulinic acid, polyhydroxyalcanoates, bacteriochlorophylls *a* and *b*, ubiquinones, and other valuable compounds. They are highly promising producers of molecular hydrogen. PNSB can be cultivated in organic waste waters, such as wastes after fermentation. In most cases, wastes mainly contain acetic acid. Therefore, understanding the anaplerotic pathways in PNSB is crucial for their potential application as producers of biofuels. The present review addresses the recent data on presence and diversity of anaplerotic pathways in PNSB and describes different classifications of these pathways.

## 1. Introduction

In most chemotrophic, aerobic, and facultative anaerobic bacteria, the central carbon metabolism uses the following pathways [1]: tricarboxylic acid cycle (TCA cycle), glycolysis (or similar pathways, e.g., the Entner–Doudoroff pathway), as well as gluconeogenesis and the pentose phosphate pathway [2].

These pathways take part in catabolism and anabolism. Sugars are oxidized via a glycolytic pathway (and similar reactions) with pyruvate (PA) production. The reversible pyruvate-oxidoreductase oxidizes PA into acetyl-CoA and CO_2_. The TCA cycle then oxidizes acetyl-CoA, some organic acids, and acetyl-CoA-metabolized substrates into CO_2_. The reducing equivalents derived from the degradation of carbohydrates and TCA cycle substrates initiate the ATP synthesis via aerobic and anaerobic respiration. This is the catabolic function of carbon metabolism pathways.

**Figure 1:**
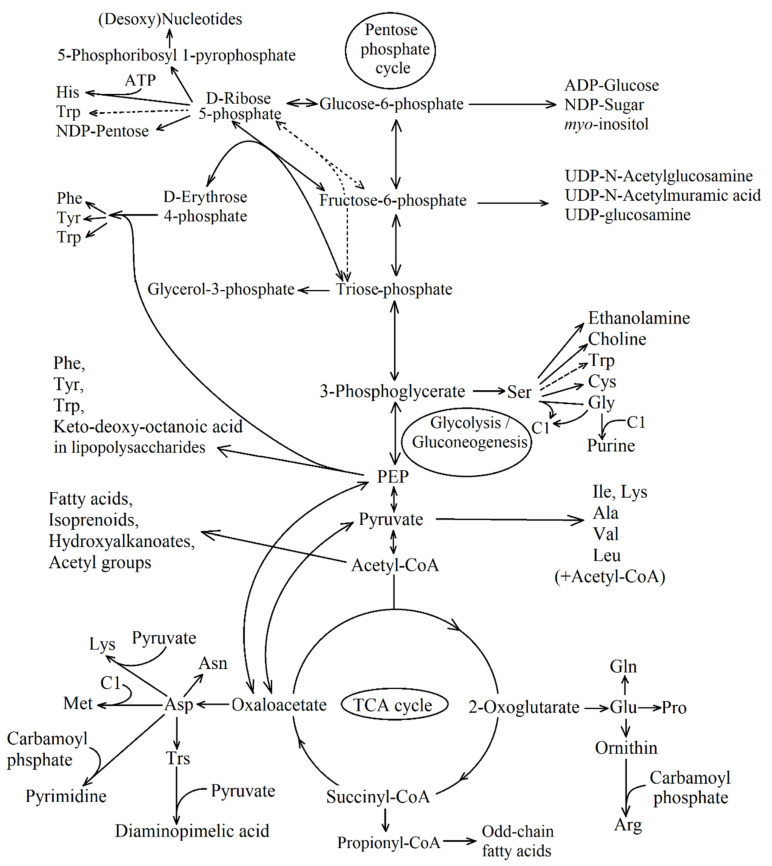
Synthesis of building blocks from the main intermediate products of carbon metabolism. C1—C1 fragments bound to tetrahydrofolate.

The intermediates of these crucial metabolic pathways include 12 major precursor metabolites that are used to synthesize 70–100 cell building blocks (Figure 1). The most important ones are L-amino acids, nucleotides, activated sugars, and fatty acids. Thus, the biosynthesis stems from the degradation reactions.

In purple bacteria, TCA cycle is active during the phototrophic anaerobic growth and dark aerobic respiration. In case of anaerobic phototrophic growth, when the bulk of ATP is a product of the light-dependent electron transport, the TCA cycle performs cellular biosynthesis and participates in the constructive metabolism (by converting one group of central building blocks into another for further biosynthesis, Figure 1). In addition, the carbon dioxide released during the oxidation of substrates in the cycle can act as C1-units for synthesizing certain groups of compounds. For example, the photoheterotrophic growth of *Rps. palustris* using acetate transforms 22% of acetate into CO_2_, 67% of which is then fixed into the cellular material via ribulose bisphosphate carboxylase/oxygenase (Rubisco) [3]. 

TCA cycle activity is varied depending on the carbon substrate type of the medium [4] and on the physiological state of the culture. It effectively acts as a roundabout on a busy highway, redistributing molecules in a similar way to cars to avoid traffic congestion or disruption [5]. Carboxylases are involved in this mutual conversion (reconstruction), e.g., amino/fatty acids being reconstructed into the carbohydrate precursors by the TCA cycle reactions [1]. Cells can assimilate acetate as a sole organic substrate in the case of anaplerotic pathways replenishing the oxaloacetate (OAA) pool.

The PNSB produce polyhydroxyalcanoates, a-aminolevuline acid, bacteriochlorophylls *a* and *b* and molecular hydrogen. During the anoxygenic photosynthesis, PNSB use organic acids as electron donors. Taking into account the fact that many organic waste waters contain acetate as the key organic acid, scrutinizing anaplerotic pathways in PNSB is essential.

Here, we overview the latest data on the presence and diversity of anaplerotic pathways in PNSB, and describe different classifications of these pathways.

## 2. Part 1: TCA Cycle Replenishing Pathways

For cells using acetate as a sole organic substrate, the TCA cycle should be sustainable. An anaplerotic pathway replenishing the oxaloacetic pool should function due to the outflow of the TCA cycle intermediates. Evidently, the existence of a single anaplerotic pathway is necessary and sufficient. However, there is evidence on several anaplerotic pathways functioning simultaneously in one bacterium [6,7]. Currently, several anaplerotic pathways replenishing the OAA pool are known: the glyoxylate pathway, the ethylmalonyl-CoA pathway, pyruvate synthase in combination with carboxylating enzymes [8], the citramalate cycle [9,10], and the methylaspartate cycle [11,12]. Carbon dioxide produced via the TCA cycle can be used not only in the pyruvate synthase reaction with the following PA carboxylation, but also in autotrophic CO_2_ fixation [13]. Under the conditions of bacterial growth with acetate or butyrate used as the only organic substrate, the main function of CO_2_-fixing reactions is believed to be maintaining the redox balance of the cell [14,15]. 

Currently, seven pathways of autotrophic fixation of carbon dioxide are known: the 3-hydroxypropionate bi-cycle [16,17,18]; the Calvin–Benson–Bassham cycle (CBB cycle; [19]); the reductive citric acid cycle (rTCA cycle; [20]); the 3-hydroxypropionate/4-hydroxybutyrate (HP/HB) cycle [21]; the dicarboxylate/hydroxybutyrate (DC/HB) cycle [22]; the reductive acetyl-CoA pathway [23]; and the reductive glycine pathway [24]. The possibility of involving the CO_2_-fixation pathways in the synthesis of TCA cycle intermediates is discussed below.

**Figure 2:**
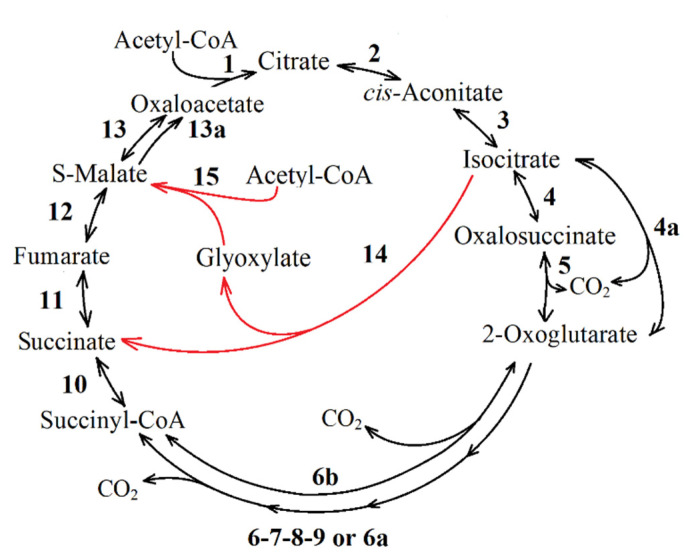
TCA cycle (reaction **1**–**13**) and glyoxylate shunt (reactions **14**, **15**). The scheme was constructed on the basis of KEGG Pathway database. **1**—citrate synthase; **2** and **3**—aconitate hydratase; **4** and **5**—isocitrate dehydrogenase; **4a**—isocitrate dehydrogenase (NAD+) or isocitrate—homoisocitrate dehydrogenase; **6** and **7**—oxoglutarate dehydrogenase (succinyl-transferring); **8**—dihydrolipoyllysine-residue succinyltransferase; **9**—dihydrolipoyl dehydrogenase; **6a**—2-oxoacid oxidoreductase (ferredoxin); **6b**—2-oxoglutarate synthase; **10**—succinyl-CoA synthetase (ADP-forming) or succinyl-CoA synthetase (GDP-forming) or succinyl-CoA:acetate CoA-transferase; **11**—fumarate reductase (quinol) or succinate dehydrogenase; **12**—fumarate hydratase; **13**—malate dehydrogenase; **13a**—malate dehydrogenase (quinone); **14**—isocitrate lyase; **15**—malate synthase.

**The glyoxylate cycle** is the most common pathway for replenishing the OAA pool in the TCA [25]. In a functional TCA cycle (Figure 2, reactions 1 to 13), the presence of this anaplerotic pathway depends on two enzymes: isocitrate lyase, ICL (EC: 4.1.3.1), and malate synthase (EC: 2.3.3.9) (Figure 2, reactions 14 and 15). The full glyoxylate cycle is a combination of glyoxylate shunt and some TCA cycle reactions, and includes reactions 1–3, 11–13, and 14, 15.

One full turn of the glyoxylate cycle produces a TCA cycle intermediate (succinate) and a precursor molecule (glyoxylate) following the malate synthesis from glyoxylate and acetyl-CoA (reactions 14, 15, Figure 2). Both of them maintain the concentration of oxaloacetate during TCA cycle intermediate outflow. Glyoxylate shunt is believed to be non-functional in anaerobic growth [26,27]. However, it is active in anaerobic bacteria, including phototrophs, provided that ferredoxin is present as a cofactor, and pyruvic acid (and α-ketoglutarate) can be produced by reductive carboxylation [28,29].

Several PNSB (*Rhodobacter sphaeroides*, renamed as *Cereibacter sphaeroides* [30]; *Rsp. gelatinosus*, renamed as *Rubrivivax gelatinosus* [31]; *Rs. rubrum*; *Phaeospirillum fulvum*, renamed as *Magnetospirillum fulvum* [30]) do not have ICL activity [6,7,32,33]. It should be noted that the domain Bacteria has been substantially re-classified (www.bacterio.net, accessed on 17 February 2020). As a result, many strains were renamed. In this review, we use the names after the authors of the publications with a new name in parentheses at the first mention. Some of them do not have malate synthase activity (*Rubrivivax gelatinosus* [33]).

In *Rba. capsulatus* [32,34,35,36,37] and *Rsp. palustris* [32], ICL is an inducible enzyme that is active in the organisms grown on the acetate or butyrate. In both bacterial species, adding C4 TCA cycle intermediates such as succinate or malate [32,38] reduces ICL activity in butyrate- or acetate-grown cultures. The synthesis of this enzyme is resumed after a period, during which the cells supposedly consume C4 substrate. Addition of lactate (metabolized through PA) does not inhibit the ICL synthesis. Furthermore, the enzyme is synthesized after switching from growth using acetate to a lactate-containing medium upon acetate depletion at least for several hours [37].

Non-deactivation of ICL in lactate presence could arise from acetyl-CoA produced from the pyruvic acid by the pyruvate dehydrogenase and pyruvate:oxidoreductase. Acetyl-CoA is then involved in TCA cycle and glyoxylate cycle reactions. Pyruvate:oxidoreductase transcripts were not found in some of the samples of phototrophically acetate-grown *Rba. capsulatus* [39]. However, pyruvate dehydrogenase was confirmed experimentally in the phototrophic cultures of *Rba. capsulatus* B10 grown with acetate and showing the active glyoxylate shunt enzymes [35]. Notably, switching from the growth using TCA cycle intermediates to the growth on acetate as the only source of carbon induced a long lag period in *Rba. capsulatus* St. Luis [34]. No lag of this kind was observed when lactate-grown *Rba. capsulatus* B10 culture were shifted to medium with acetate [40].

ICL^+^ bacteria *Rba. capsulatus* [33,37,41] similar to non-phototrophic bacterium *Paracoccus denitrificans* [42] were able to use acetate as the only organic substrate for the phototrophic growth while lacking an active glyoxylate cycle. For *Rba. capsulatus*, this happens during transience from a single lactate to a single acetate assimilation if the inoculum was more than 5 h in the stationary growth phase after lactate exhaustion [37]. Both bacteria can use the ethylmalonyl-CoA pathway [39,42] when ICL is inactive. The purple non-sulfur ICL^+^ bacterium *Rps. palustris* has been reported to exhibit TCA cycle and glyoxylate cycle enzyme activity when growing in the acetate medium [43]. TCA cycle and glyoxylate cycle enzymes as well as Rubisco were found to be fully present in cell-free extracts of *Rps. palustris* after growing under chemoheterotrophic, photoheterotrophic and photolithotrophic conditions [43]. The glyoxylate cycle had the highest ICL activity after photoheterotrophic growth with acetate. The photo- and chemoheterotrophic growth using the malate medium halved this activity. The enzyme activity was not suppressed during photolitotrophic growth with thiosulfate. At present, it is clear that ICL^−^ and ICL^+^ PNSB without ICL activity possess other anaplerotic pathways.

**Figure 3:**
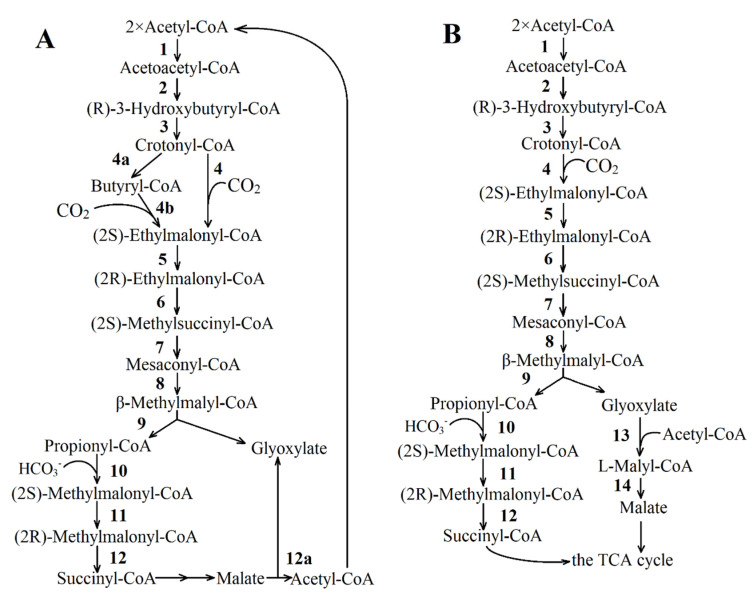
Ethylmalonyl-CoA pathway. (**A**) *Methylotrophs* [44]. (**B**) *Rba sphaeroides* [8]. KEGG Pathway database: **1**—acetyl-CoA C-acetyltransferase; **2**—acetoacetyl-CoA reductase; **3**—3-hydroxybutyryl-CoA dehydratase; **4**—crotonyl-CoA carboxylase/reductase; **4a**—trans-2-enoyl-CoA reductase (NAD+); **4b**—no date; **5**—methylmalonyl-CoA/ethylmalonyl-CoA epimerase; **6**—ethylmalonyl-CoA mutase; **7**—(2*S*)-methylsuccinyl-CoA dehydrogenase; **8**—2-methylfumaryl-CoA hydratase; **9**—malyl-CoA/(*S*)-citramalyl-CoA lyase; **10**—propionyl-CoA carboxylase; **11**—methylmalonyl-CoA/ethylmalonyl-CoA epimerase; **12**—methylmalonyl-CoA mutase; **12a**—malate synthase; **13**—malyl-CoA/(*S*)-citramalyl-CoA lyase; **14**—(3*S*)-malyl-CoA thioesterase.

**The ethylmalonyl-CoA pathway** was discovered in some methylotrophs [44,45,46]. This cycle converts two acetyl-CoA and two CO_2_ molecules into two glyoxylate molecules, CoA  and H^+^ (Figure 3A). Glyoxylate can further be serine-converted into PEP by assimilating one carbon unit via N5, N10-methylene tetrahydrofolate [47]. Figure 3B shows an alternative modification of this pathway characteristic for the purple non-sulfur bacterium *Rba. sphaeroides* [48]. Unlike the pathway of *Methylorubrum extorquens* AM1, renamed as *Methylorubrum extorquens* [49], the *Rba. sphaeroides* variant of the ethylmalonyl-CoA pathway condenses the produced glyoxylate with acetyl-CoA to produce malyl-CoA, which is then hydrolyzed into malate and CoA. Thus, the ethylmalonyl-CoA cycle of the purple non-sulfur bacterium produces one malate molecule and one succinyl-CoA molecule from three acetyl-CoA and two CO_2_ molecules.

This pathway is active in the ICL^−^PNSB, *Phaeobacter inhibens* strain BS107 after the dark aerobic growth [50]; *Rs. rubrum* [7,51], and in the ICL^+^ bacteria *Rba. capsulatus* [39,41]; *P. denitrificans* [42]). Genomic analysis did not reveal this pathway in the ICL^+^ bacterium *Rps. palustris* [13]. The enzymes of ethylmalonyl-CoA pathway were shown to be expressed constitutively while being grown on various sources of carbon, which distinguishes it from the glyoxylate cycle, being specifically acetate-induced [7,42]. This might be due to the fact that the ethylmalonyl-CoA pathway allows for direct absorption of propionate and several dicarboxylic acids and is interrelated with the synthesis of storage compounds (polyhydroxyalkanoates or PHAs); it can therefore be used by the bacterium as a multipurpose pathway for different environments [42].

The purple non-sulfur bacterium *Rs. rubrum* strain S1H uses the ethylmalonyl-CoA pathway as the main pathway for assimilating acetate during photoheterotrophic growth with acetate as the only organic source. Notably, the acetate-grown cultures contained detectable enzymes of the CBB cycle, pyruvate synthase/pyruvate:oxidoreductase, and enzymes of citramalate synthase-involving pathway [7,51]. The authors found that a mutant *Rs. rubrum* strain S1H with a knocked-out key gene of the ethylmalonyl-CoA pathway (crotonyl-CoA reductase/carboxylase) grew aerobically or anaerobically using the succinate medium [51]. However, this mutant could not grow using acetate as the sole organic source regardless of the bicarbonate’s presence in the medium underphotoheterotrophic conditions. In addition, the growth of this mutant was inhibited under the chemoheterotrophic aerobic conditions with acetate.

The genomic analysis shows that the ethylmalonyl-CoA pathway is the main anaplerotic pathway for the genus *Paracoccus*, allowing the replenishment of the oxaloacetate pool [42]. Of note is that the key genes of two anaplerotic pathways (the glyoxylate cycle and the ethylmalonyl-CoA pathway) were found in *P. denitrificans* when the cells grew with acetate as the only organic substrate [42]. The quantities of these enzymes were different in different growth phases. However, neither the ICL gene deletion nor that of the crotonyl-CoA reductase/carboxylase gene were lethal for the acetate assimilation by this bacterium. Each of the anaplerotic pathways was found to give the organism a specific advantage. The mutants’ analysis revealed that the ethylmalonyl-CoA pathway in *P*. *denitrificans* apparently improves the biomass yield, whilst the glyoxylate cycle enables a faster growth on a medium with acetate being the only organic substrate. Utilizing the succinate, both strains with deletions grew similarly to the wild type. However, the strain without ethylmalonyl-CoA pathway showed a longer lag phase.

The authors [42] compared the translational activity of *ccr* and *icl* genes (encoding crotonyl-CoA reductase/carboxylase and ICL, respectively) in *P*. *denitrificans* grown with succinate or acetate. In the bacterial cells grown with succinate, only the *ccr* preserved their quantity at the low basal level.

In *Rba. capsulatus*, which also has both the glyoxylate cycle and ethylmalonyl-CoA pathway genes, the ICL activity had patterns similar to the ccr in *P*. *denitrificans* when growing on acetate at an uncontrolled pH [40]. However, when the optimal pH level was maintained, the ICL activity in the acetate culture increased similarly to those of *icl* in *P*. *denitrificans* [37].

The recent data show that this particular cycle can be combined in the ICL^+^ PNSB as in other bacteria with the glyoxylate cycle.

**Figure 4:**
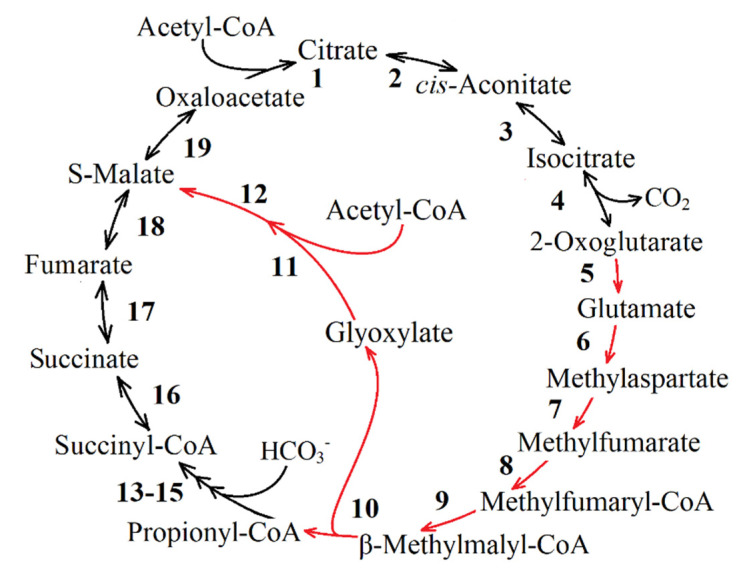
Methylaspartate cycle. The scheme was constructed on the basis of the KEGG Pathway database and [11]: reaction of TCA cycle **1**–**4**, **16**–**19**; the key reactions of the anaplerotic pathways are shown in red **5**–**12** (**5**—glutamate dehydrogenase; **6**—methylaspartate mutase; **7**—methylaspartate ammonia-lyase **8**—succinyl-CoA:mesaconate CoA transferase; **9**—2-methylfumaryl-CoA hydratase; **10**—malyl-CoA/(*S*)-citramalyl-CoA lyase; **11**—malyl-CoA/(*S*)-citramalyl-CoA lyase; **12**—(3*S*)-malyl-CoA thioesterase); reaction of methylmalonyl-CoA pathway (**13**—propionyl-CoA carboxylase; **14**—methylmalonyl-CoA/ethylmalonyl-CoA epimerase; **15**—methylmalonyl-CoA mutase).

**The methylaspartate cycle [11,52,53]**. The reaction sequence of this metabolic pathway was first demonstrated for the haloarchaeon *Haloarcula marismortui* (Figure 4). The methylaspartate cycle was elucidated combining proteomics and the enzyme activities measurements with the *H. marismortui* [11]. Using the gene deletion mutants of *H. hispanica*, enzyme assays and metabolite analysis, the authors bridged the knowledge gaps by an unambiguous identification of the genes encoding all characteristic enzymes of the cycle [54].

In the methylaspartate cycle, acetyl-CoA is transformed to glutamate via reactions of the TCA cycle and glutamate dehydrogenase. The rearrangement of glutamate into methylaspartate and its subsequent deamination leads to mesaconate (2-methylfumarate) production. Mesaconate is then activated to mesaconyl-CoA (2-methylfumaryl-CoA), which is hydrated to β-methylmalyl-CoA, and β-methylmalyl-CoA is finally cleaved to propionyl-CoA and glyoxylate. Propionyl-CoA carboxylation leads to the methylmalonyl-CoA and subsequently to succinyl-CoA production, thus completing the cycle, whereas the condensation of glyoxylate with another molecule of acetyl-CoA yields malate, the final product of the methylaspartate cycle [11]. The enzymes of this pathway are discussed below.

A functioning methylaspartate cycle is expected to require a high intracellular glutamate concentration, because the affinity for substrate (Km) of methylaspartate ammonia-lyase for methylaspartate in *H. marismortui* was determined as 26 ± 5 mM, and the equilibrium of the preceding glutamate mutase reaction is not on the side of methylaspartate (Km = 0.093 at 30 °C) [55]. Indeed, the cytoplasmic glutamate concentration in the acetate-grown *H. marismortui* cells was 35 ± 5 mM (compared with 6 ± 1 mM in acetate-grown, ICL^+^ *H. volcanii*) [11].

It was shown that methyaspartate and glyoxylate cycles are evenly distributed in haloarchaea [54]. Interestingly, 83% of the species using the methylaspartate cycle also possess the genes for polyhydroxyalkanoate biosynthesis, whereas only 34% of the species with the glyoxylate cycle are capable of synthesizing this storage compound. The authors suggest that the methylaspartate cycle is shaped for the polyhydroxyalkanoate utilization during carbon starvation, whereas the glyoxylate cycle is probably adapted for growing on substrates metabolized via acetyl-CoA. Interestingly, some haloarchaea possess genes for both cycles in the genome [54]. This suggests the possibility that the pathways may be adapted to fulfill various functions or allow microorganisms to adapt to a fast-changing environment.

There has been no evidence in favor of the methylaspartate cycle functioning in PNSB. Analysis of genomes of *Rba. capsulatus* SB1003 and 9 strains of *Rsp. palustris* did not reveal any genes necessary for this pathway in bacteria [13,39].

**Figure 5:**
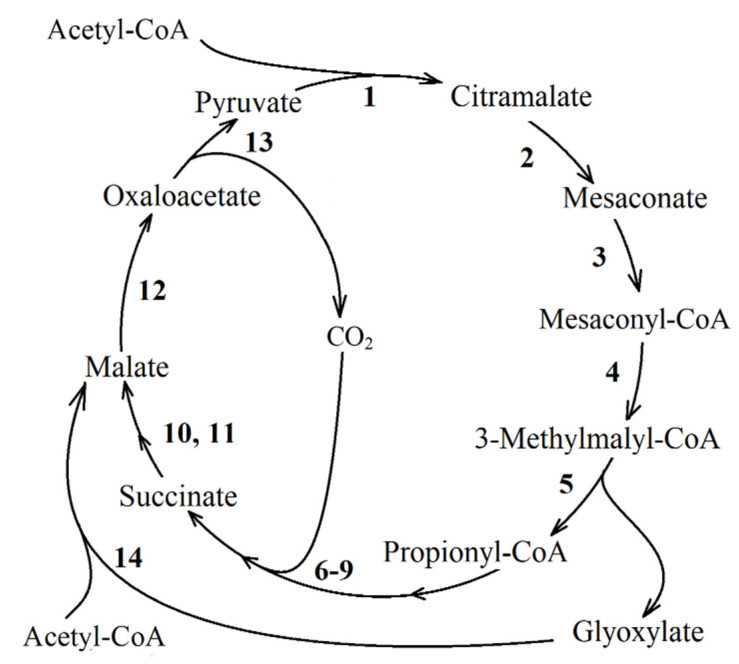
Citramalate cycle [56]. **1**—citramalate synthase; **2**—mesaconase; **3**—mesaconate CoA-transferase; **4**—2-methylfumaryl-CoA hydratase; **5**—3-methylmalyl-CoA lyase; **6**—propionyl-CoA carboxylase; **7**—methylmalonyl-CoA epimerase; **8**—methylmalonyl-CoA mutase; **9**—succinyl-CoA synthetase or succinyl-CoA:acetate CoA-transferase; **10**—fumarate reductase (quinol) or succinate dehydrogenase; **11**—fumarate hydratase; **12**—malate dehydrogenase; **13**—oxaloacetate decarboxylase; **14**—malate synthase reaction.

**The citramalate cycle** (Figure 5) was found in the purple non-sulfur bacterium *Rsp. rubrum* [9] and later proposed for *Rba. sphaeroides* 2R [56]. In this pathway, acetyl-CoA and PA are condensed into citramalate, which is converted to mesaconate. The latter is converted to mesaconyl-CoA, which is further converted to methylmalyl-CoA. Methylmalyl-CoA is cleaved into glyoxylate and propionyl-CoA. Glyoxylate can be involved in the TCA cycle through a malate synthase reaction or, alternatively, used for the biosynthetic needs of the cells grown with C4-substrates. In turn, propionyl-CoA is converted into PA during a series of reactions with the succinyl-CoA formation step, which completes the cycle.

Leroy [7] reported that in the *Rs. rubrum* S1H, citramalate formed from PA and acetyl-CoA may not be a metabolite of the citramalate cycle, but an intermediate compound of the branched chain amino acid biosynthesis/degradation pathway (isoleucine and valine, ILV synthesis pathway) or a novel alternative anaplerotic acetate assimilation pathway through PA and part of the ILV biosynthesis pathway [7,51,57]. These pathways are activated during the acetate assimilation. It should be noted that once grown with the substrates such as malate or fumarate, the citramalate-dependent route of isoleucine synthesis does not function in *Rs. rubrum* even in mutant strains with an inactivated CBB cycle [58]. In this case, the redox balance is maintained by the threonine-dependent synthesis pathway ILV. The authors detected the reversal of some TCA cycle enzymes, which carried the reductive flux from malate or fumarate to α-ketoglutarate. This pathway and the reductive synthesis of amino acids derived from α-ketoglutarate are likely to be important for the electron balance. This suggestion is supported by the fact that adding α-ketoglutarate-derived amino acids to the medium prevented the *Rs. rubrum* CBB cycle mutant growth when a terminal electron acceptor was absent [58]. The flux estimates also suggested that the CBB cycle mutant preferentially synthesized isoleucine using the reductive threonine-dependent pathway instead of a less-reductive citramalate-dependent pathway. Interestingly, unlike a similar mutant strain of *Rps. palustris* with an inactivated CBB cycle, mutant *Rs. rubrum* was not capable of growing with succinate.

Later, additional reactions of conversion of acetyl-CoA to propionyl-CoA, involving pyruvate synthase and part of the pathway of biosynthesis/degradation of IVL, were revealed [59]. The authors called this pathway methylbutanoyl-CoA. The ethylmalonyl-CoA pathway and methylbutanoyl-CoA pathways appear to be simultaneously used by *Rs. rubrum*.

The citramalate pathway has no potential for functioning in the studied strains *Rps. palustris* [13]. The possibilities of the citramalate cycle functioning in *Rba. capsulatus* have not been clearly defined, since the data on the presence of enzymes catalyzing 2-methylfumarate formation from (*S*)-citramalate and (*S*)-citramalyl-CoA conversion into (*S*)-citramalate are not available [39]. Additional research is necessary to clarify the distribution of this cycle in purple bacteria.

**The reversiblepyruvate:ferredoxin oxidoreductase (orpyruvate synthase).** In strict anaerobes, acetyl-CoA is generally transformed into PA by pyruvate:ferredoxin oxidoreductase, the same enzyme that functions in the reversed TCA cycle (rTCA cycle), the DC/HB cycle, and the reductive acetyl-CoA pathway [60]. With no active glyoxylate cycle reported, green sulfur bacteria and heliobacteria use pyruvate synthase for acetate assimilation [8]. Pyruvate synthase synthesizes PA from CO_2_ and acetyl-CoA produced through acetate uptake and the rTCA cycle during mixotrophic growth of green sulfur bacteria [20,61,62]. This PA formation pathway in combination with carboxylating enzymes can also function in facultative anaerobes growing in the absence of oxygen [8] since the enzyme is sensitive to oxygen. It has been shown that pyruvate:oxidoreductase (NifJ) can be used in *Rs. rubrum* S1H for direct absorption of acetyl-CoA through PA [7].

Since this enzyme is sensitive to oxygen, the acetate assimilation via the above route is not possible under the aerobic conditions; thus, alternative pathways exist in (micro)aerobic organisms. Furdui and Ragsdale [63] measured a significant carboxylating activity under physiological concentrations and showed that the reaction rate depends on the concentration of acetyl-CoA and PA only.

It has been shown that PA could be synthesized from acetate in nine examined strains of *Rps. palustris* via the reaction of pyruvate:ferredoxin oxidoreductase. Meanwhile, this pathway is transcriptionally active in acetate cultures of *Rps. palustris* CGA009 and some acetate cultures of *Rba. capsulatus* [13]. Whereas PA synthesis from acetyl-CoA and formate is possible in *Rps. palustris* strains BisB18 and BisA53, the genes coding proteins for these reactions were not expressed in the *Rba. capsulatus* cultures grown with acetate.

**The CBB cycle.** In this cycle, carbon dioxide is fixed to form C6-compounds. It can serve as a substrate for carboxylases; 3-PGA formed in this metabolic pathway under the glycolytic pathway enzymes can be further converted into PEP and PA. They can be subsequently carboxylated to malate or oxaloacetate the replenishing oxaloacetate pool of TCA cycle. The Rubisco, phosphoribulokinase and sedoheptulose bisphosphatase [64] are unique, i.e., functioning only in this cycle.

The key enzyme that fixes CO_2_ in the CBB cycle is Rubisco. Currently, different forms of Rubisco have been found in microorganisms. Four main forms of Rubisco are distinguished [65]. Rubiscos of forms I, II, and III exhibit carboxylase and oxygenase activity, but for potentially different physiological purposes. Form III is distinguished into a separate category, since it is found only in archaea, having a unique ancient evolutionary history. Proteins of form IV include Rubisco-like proteins (Rubisco-like protein, RLP), catalyzing reactions that are important for sulfur metabolism. In many organisms, the function of RLP is unknown.

It should be noted that part of genes annotated as Rubisco subunits in the recently sequenced *Rps. palustris* strains display high protein sequence identity with the Rubisco-like proteins *rlp1* and *rlp2* [13]. *Rba. sphaeroides* has two forms of Rubisco. Form I consists of eight large and eight small subunits that encode *rbcL* and *rbcS* genes, respectively. Form II is a hexamer that consists only of large subunits encoded by either the *rbcL* gene or the *rbcR* gene [66]. *Rba. capsulatus* [67] and *Rps. palustris* [68] also have two forms of Rubisco. They are encoded by the genes *cbbLS* (form I Rubisco) and *cbbM* (form II Rubisco). Unlike these bacteria, *Rsp. rubrum* has only form II Rubisco, which consists of two subunits encoded by the *cbbM* gene [66,69]. Surprisingly, this protein was able to function not only as Rubisco, but also as an enolase in the methionine utilization pathway (a methionine salvage pathway, MSP) anaerobic conditions [70,71].

*Rba. sphaeroides*, *Rba. capsulatus*, and *Rps. palustris* genes of form I and form II Rubisco are located in *cbbI* and *cbbII* operons, respectively [69]. The regulation of *cbb* operons of these bacteria has been thoroughly studied [66,68,72,73,74,75,76]. The transcription of *cbb* operons has been shown to be activated by the Lys-R-type transcription regulator (*CbbR* [74,77]. This regulator may need ribulose-1,5-bisphosphate or its derivatives as co-inductors [78,79,80,81]. In addition, the expression of *cbb* operons is also regulated by the two-component global regulatory system RegB/RegA, which regulates the processes of nitrogen fixation, hydrogen metabolism, and energy production in *Rba. capsulatus* and *Rba. sphaeroides* [76,82,83,84,85,86,87,88]. *Rps. palustris* has no regulatory system RegB/RegA. Instead, three proteins of the CbbRRS system act as a transduction signal system that regulates the transcription of *cbb* operons [75]. In turn, in *Rsp. Rubrum*, both RegB/RegA and CbbRRS systems are absent. In addition, in *Rsp. Rubrum*, the gene of *cbbM* is not located in one operon with other *cbb* genes, unlike other organisms [79,89,90]. In this bacterium, the main regulator of *cbb* operon (cbbEFPT) is the CbbR—positive transcription regulator [91].

Mutant strains of *Rba. sphaeroides* that synthesize either form I or form II Rubisco grew in the photoautotrophic conditions, although slower than the wild type [73]. The phototrophic growth RubisCO gene-deletion strains of the *Rs. rubrum* and *Rba. sphaeroides* was observed when CO_2_ was the only carbon source using inorganic electron donors less reduced than molecular hydrogen, i.e., thiosulfate or sulfide [92,93]. The authors suggested that there are two independent CO_2_ fixation pathways that support photolithoautotrophic growth in purple non-sulfur photosynthetic bacteria.

Rubisco can catalyze a reaction of ribulose-1,5-bisphosphate and molecular oxygen (O_2_) instead of carbon dioxide (CO_2_) [94]. This explains the release of glycolate into the medium in the presence of molecular oxygen [94]. In cyanobacteria and algae, glycolate is either released into the medium or oxidized by the enzyme glycolate oxidase to glyoxylate, which can be converted to glycerate through the serine pathway [94]. In the glycolate pathway, one molecule of PGA is formed from two molecules of glycolate, while a molecule of CO_2_ is released. Thus, during the functioning of Rubisco as oxygenase, oxygen uptake and carbon dioxide release occur. The combination of the light-dependent absorption of oxygen and the release of CO_2_ is commonly called photorespiration [64]. However, in most purple bacteria, oxygen has an inhibitory effect on both Rubisco activity and its synthesis [66].

The synthesis and activity of Rubisco depends on the concentration of CO_2_ in the medium: the amount of enzyme in cells increases with an increase in CO_2_ [95]. The level of the CBB cycle enzymes activity in PNSB is different under different growth conditions and decreases in the presence of organic substrates [96,97]. The activity of this pathway is reduced in cells after the transition from the phototrophic growth using succinate to medium with acetate [7]. This may be due to the outflow of CO_2_ to the synthesis of other significant intermediates from the acetyl-CoA (for example, carboxylation of acetyl-CoA with reversible pyruvate: oxidoreductase, as well as to the steps of carboxylation in the ethylmalonyl-CoA pathway). In some cases, PNSB fix significant amounts of CO_2_ in the CBB cycle regardless of the organic compounds. For example, 22% of acetate carbon is converted to carbon dioxide and 67% of this carbon dioxide is fixed in the cell material through Rubisco during photoheterotrophic growth of the *Rps. palustris* using acetate medium [3]. Rubisco activity of *Rps. palustris* was repressed by chemoheterotrophic growth but was not decreased during photoheterotrophic growth [43]. It has also been demonstrated that the enzyme level of Form I increased significantly during photoautotrophic growth or during growth on a very reduced substrate such as butyrate in both *Rba. sphaeroides* [98] and *Rba. capsulatus* [99]. The CBB cycle is necessary during photoheterotrophic growth with succinate, malate, fumarate or acetate to maintain a pool of oxidized electron carriers. *Rs. rubrum*, *Rs. fulvum*, renamed as *Magnetospirillum fulvum* [30,100,101] and *Rps. palustris* CBB cycle phosphoribulokinase mutants that cannot produce ribulose-1,5-bisphosphate demonstrate an inability to grow photoheterotrophically on succinate unless an electron acceptor is provided or H_2_ production is permitted [100]. CBB cycle mutants of *Rs. rubrum*, but not of *Rps. palustris*, grew photoheterotrophically on malate [100] or fumarate [55] without electron acceptors or H_2_ production.

Wang D. and coauthors suggested an alternative model wherein disrupting Rubisco activity prevents photoheterotrophic growth due to the accumulation of toxic ribulose-1,5-bisphosphate [91]. It was later demonstrated that the CBB cycle is still needed to oxidize electron carriers even in the absence of toxic ribulose-1,5-bisphosphate [100]. Considering available data, Rubisco regulation in PNSB is connected with CO_2_ fixation, or with the maintenance of the redox balance in cells but not with TCA cycle activity.

**The reductive TCA cycle**. Some rTCA cycle reactions can be considered as anaplerotic pathways replenishing TCA intermediates. They start from the reductive carboxylation of acetyl-CoA to PA catalyzed by ferredoxin-dependent pyruvate synthase. PA is converted to oxaloacetate [20] or PEP and further to oxaloacetate [102]. In PNSB with a complete set of TCA cycle enzymes, the presence of active pyruvate synthase (or pyruvate synthase and PEP synthase) in combination with carboxylating enzymes is sufficient to replenish the pool of its intermediates during the photoassimilation of acetate. So, the activity of pyruvate synthase (and PA or PEP carboxylatyng enzyme) as a part of rTCA could be considered as anaplerotic pathways replenishing the oxaloacetate pool. The same can be said about another pathway of autotrophic fixation of carbon dioxide—the dicarboxylate/hydroxybutyrate cycle [22] and the reductive glycine pathway [24].

**Figure 6:**
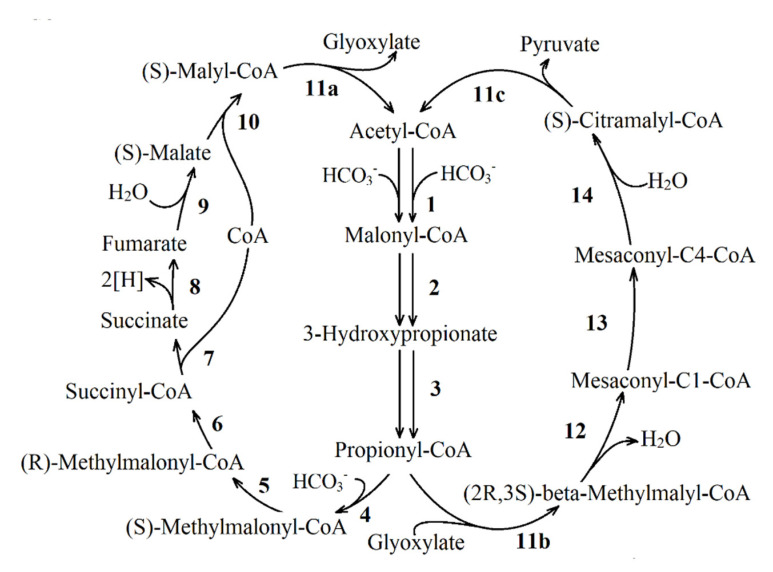
The 3-hydroxypropionate bi-cycle in *C. aurantiacus* [6]. **1**—acetyl-CoA carboxylase; **2**—malonyl-CoA reductase; **3**—propionyl-CoA synthase; **4**—propionyl-CoA carboxylase; **5**—methylmalonyl-CoA epimerase; **6**—methylmalonyl-CoA mutase; **7/10**—succinyl-CoA:(*S*)-malate-CoA transferase; **8**—succinate dehydrogenase; **9**—fumarate hydratase; **11 a/b/c**—(*S*)-malyl-CoA/β-methylmalyl-CoA/(*S*)-citramalyl-CoA lyase; **12**—mesaconyl-C1-CoA hydratase (β-methylmalyl-CoA dehydratase); **13**—mesaconyl-CoA C1:C4 CoA transferase; **14**—mesaconyl-C4-CoA hydratase.

**Figure 7:**
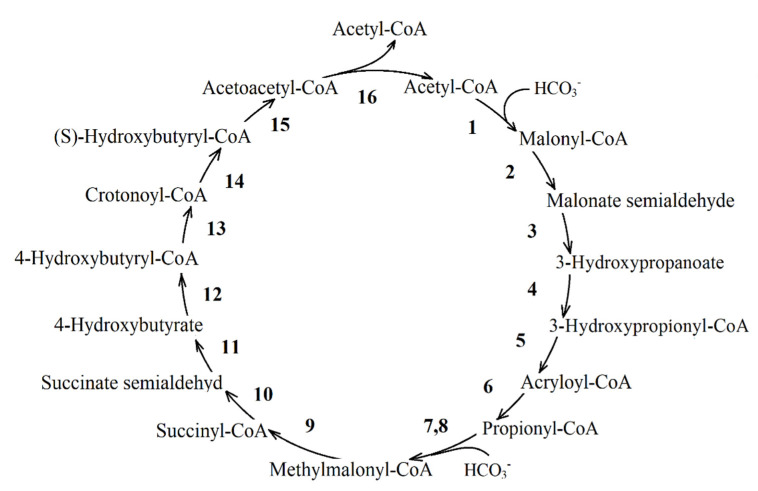
3-Hydroxypropionate/4-hydroxybutyrate cycle [21]. Reactions of the cycle are shown. Enzymes: **1**—acetyl-CoA carboxylase; **2**—malonyl-CoA reductase (NADPH); **3**—malonate semialdehyde reductase (NADPH); **4**—3-hydroxypropionyl-CoA synthetase (AMP-forming); **5**—3-hydroxypropionyl-CoA dehydratase; **6**—acryloyl-CoA reductase (NADPH); **7**—propionyl-CoA carboxylase; **8**—methylmalonyl-CoA epimerase; **9**—methylmalonyl-CoA mutase; **10**—succinyl-CoA reductase (NADPH); **11**—succinate semialdehyde reductase (NADPH); **12**—4-hydroxybutyryl-CoA synthetase (AMP-forming); **13**—4-hydroxybutyryl-CoA dehydratase; **14**—crotonyl-CoA hydratase; **15**—3-hydroxybutyryl-CoA dehydrogenase (NAD^+^); **16**—acetoacetyl-CoA β-ketothiolase.

**The 3-hydroxypropionate bi-cycle** (Figure 6) is found in the green non-sulfur bacterium *Chloroflexus aurantiacus* [16,17]. It may serve as an anaplerotic pathway, replenishing the oxaloacetate pool. This autotrophic pathway of carbon dioxide fixation consists of two cycles [18] with common reactions (reactions 1–3, Figure 6). In the first cycle, (*S*)-malyl-CoA is formed from acetyl-CoA (reactions 1–10, Figure 6). Then, (*S*)-malyl-CoA is cleaved (reaction 11a) into acetyl-CoA (it is returned to the cycle) and glyoxylate. The latter is further converted in the second cycle reactions of the 3-hydroxypropionate pathway to form (3*S*)-citramalyl-CoA (reactions 11b–14, Figure 6). The synthesized (3*S*)-citramalyl-CoA is finally cleaved to PA and acetyl-CoA. As a result of the complete turnover of 3-hydroxypropionate bi-cycle PA molecule is formed from three bicarbonate molecules. Reactions 1–6 of 3-hydroxypropionate bi-cycle (Figure 6, or in more detail reactions 1–9 in Figure 7) could replenish the TCA cycle. The step of converting propionyl-CoA to succinyl-CoA includes additional reactions discussed in Part 2.

The 3-hydroxypropionate bi-cycle is a way to coassimilate organic substrates such as glycolate, acetate, propionate, 3-hydroxypropionate, lactate, butyrate, or succinate [6]. The rate of CO_2_ assimilation by carboxylation of organic substrates might even be higher than the autotrophic carbon assimilation rate. The 3-hydroxypropionate bi-cycle makes a balanced redox state of the cell possible, since CO_2_ fixation consumes electrons. *C. aurantiacus* uses the 3-hydroxypropionate bi-cycle, together with the glyoxylate cycle, to channel organic substrates into the central carbon metabolism both in autotrophic and heterotrophic growth conditions (with and without oxygen). Only a fraction of acetate during photoheterotrophic growth on medium with acetate and bicarbonate was oxidized to CO_2_, probably in the course of the assimilation process.

The 3-hydroxypropionate bi-cycle of the *Chloroflexi* phylum evolved late in the Earth’s history as a result of a series of horizontal gene transfer events. This explains the lack of geological evidence for this pathway based on the carbon isotope record [103]. Data on the activity of this cycle in PNSB are not available. This cycle does not take part in OAA pool replenishment in *Rba. capsulatus* [39]. Genes of virtually all enzymes participating in this pathway are present in the genomes of some strains *Rps. palustris* [13]. However, additional research to confirm the presence of this metabolic pathway in *Rps. palustris* is necessary.

**The 3-hydroxypropionate/4-hydroxybutyrate cycle** was found in *Metallosphaera sedula* [21]. This metabolic pathway (Figure 7) begins with the carboxylation reaction of acetyl-CoA to malonyl-CoA. Some intermediates and carboxylation reactions of this pathway coincide with the reactions of the 3-hydroxypropionate cycle.

One complete turn of this cycle under the autotrophic growth leads to the formation of two acetyl-CoA molecules. One molecule is recycled in the biosynthesis of the cell material. In the presence of exogenous acetate, the functioning of enzymes of the 3-hydroxypropionate bi-cycle (Figure 7; reactions 1–9) is sufficient to replenish the pool of TCA cycle intermediates. Data on the activity of this full cycle in PNSB are not available.

## 3. Part 2: Integration of TCA Cycle Replenishing Pathways. Separation of Them on the Basis of Main Metabolites

Biochemical research has recently shown a surprising diversity in the central carbon metabolism. Amounting evidence indicates that anaplerotic pathways function in complex. This provides bacteria with additional advantages, such as an increased growth rate or higher efficiency of substrate utilization. For example, purple non-sulfur bacterium *Rba. capsulatus* does not have ICL activity during the shift from lactate to acetate medium [37]. Most probably, the growth is supported by the function of the ethylmalonyl-CoA pathway [39,41]. Once induced ICL in addition to the ethylmalonyl-CoA pathway boosts the growth rate of bacterium [37]. ICL^−^ purple non sulfur bacterium in addition to the citramalate cycle [9] or alternative isoleucine-valine pathway [7] have an ethylmalonyl-CoA pathway and pyruvate:oxidoreductase which can be used for PA synthesis from acetyl CoA [7]. The presence of several anaplerotic pathways gives higher flexibility in TCA intermediates replenishment and redox balance regulation in *Rs. rubrum*.

### 3.1. Four Groups of Pathways

**Figure 8:**
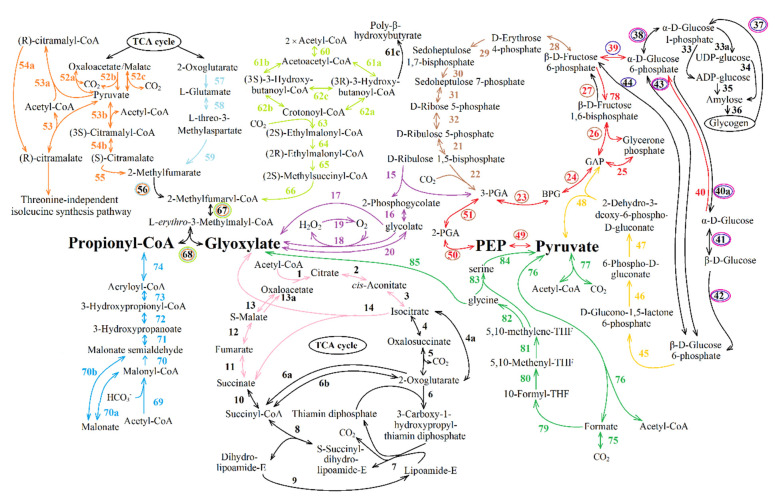
Pathways of four central precursors of the TCA cycle intermediates synthesized: **group (I)** includes pathways of glyoxylate formation (some of the reactions of the glyoxylate cycle (**reactions 1–3, 14, 10–13; here and below the color of the text corresponds to the color of reactions on figure**); pathways involving Rubisco oxygenase activity (**reactions 15, 16, and 17 or 18–19 or 20**) and the glyoxylate formation pathway from glycine formed at one stage of the reductive glycine pathway (**reactions 75,79–82,85**); **group (II)** includes pathways of propionyl-CoA and glyoxylate simultaneous formation (Part of methylaspartate cycle reactions (**reactions 57–59, 56, 67–68**), some citramalate cycle reactions (**reactions****52, 53b, 54b, 55, 56, 67–68**) and the ethylmalonyl-CoA pathway (**reactions 60, 61a-62a or 61b-62b, 63–68**); **group (III)** includes pathways of propionyl-CoA synthesis (some of the reactions of 3-hydroxypropionate and of 3-hydroxypropionate/4-hydroxybutyrate cycles (reactions **69, 70 or 70a–70b, 71–74**); **group (IV)** includes with PA/PEP formation pathways. This group consists of subgroups A, B, and C. **Subgroup A** combines two pathways of PA formation from exogenous acetate and CO_2_: the first pathway involves PA formation through pyruvate oxidoreductase (**reactions 77**); the second pathway involves PA formation involving reversible formate dehydrogenase and reversible formate-C-acetyltransferase (**reactions 75, 76**); **Subgroup B** includes the pathway of PA/PEP formation from stored carbohydrates (glycogen): the Entner–Doudoroff pathway (**reactions 45–48**) and the Embden–Meyerhof–Parnas pathway (**reactions 40, 39, 27/78, 26, 25, 24, 23, 51, 50, 49**); reactions of glycogen decomposition to β-d-fructose-6-phosphate (**reactions 37–38, 39 (or 40a, 41–43, or 43–44)**) and to β-d-Glucose-6-phosphate (**reactions 37, 38, 40a, 41–42 (or 43)**). **Subgroup C** includes the PA/PEP formation pathway from CO_2_: the PA/PEP formation pathway (through serine) from glycine synthesized in the reductive glycine pathway (**reaction 75,79–84**); and PEP/PA formation from the CBB cycle intermediates (**reactions 21–32, 51, 50, 49**). The scheme is based on the KEGG Pathway database and pertinent literature analyzed. The functional numbers of enzymes and the description of each function according to KEGG Orthology are presented in the tables under the same number as the catalyzed reaction illustrated in this figure.

**Figure 9:**
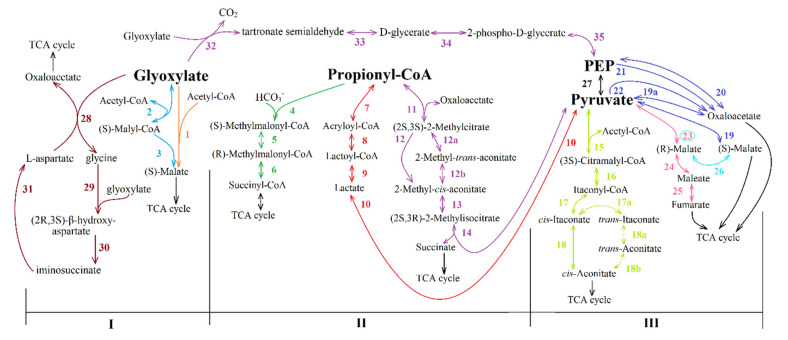
Pathways of glyoxylate, propionyl-CoA and PA/PEP conversion into TCA cycle components. I. Pathways of glyoxylate conversion: the conversion into malate by two malate synthases differing in stability and activators. (EC: 2.3.3.9)—**reaction 1**; malyl-CoA/(*S*)-citramalyl-CoA-lyase (EC: 4.1.3.24/4.1.3.25) and (3*S*)-malyl-CoA-thioesterase. (EC: 3.1.2.30)—**reactions 2–3**; pathways of glyoxylate conversion through the part of glycerate pathway (**reactions 32–35 **simultaneously with pathways of PA/PEP conversion into TCA cycle intermediates); pathways of glyoxylate conversion through the β-hydroxyaspartate cycle (**reactions 28–31,28**). II. Pathways of propionyl-CoA conversion into succinyl-CoA or PA: the methylmalonyl-CoA pathway (**reactions 4–6**); the methylcitrate pathway (**reactions 11, 12 (or 12a–12b), 13, 14**); and the pathway of propionyl-CoA oxidation to PA via lactate (**reactions 7–10**). III. Pathways of PA/PEP conversion into TCA cycle intermediates: PA/PEP carboxylating enzymes (**reactions 19, 19a, 20–22**); the pathway of fumarate (**reactions 23–25**) or (*S*)-malate (**reactions 23, 26**) formation from PA; *cis*-aconitate formation from PA and acetyl-CoA (**reactions 15–17, 18 (or 17a, 18a, 18b)**). The scheme is based on the KEGG Pathway database and pertinent literature analyzed. The functional numbers of enzymes and the description of each function according to KEGG Orthology are presented in the tables under the same number as the catalyzed reaction illustrated in this figure.

The data on the currently known enzymes show that anaplerotic pathways of replenishing the OAA pool have both common and variable chains of reactions [13,39]. The TCA cycle intermediates are formed through the stage of one of the four main metabolites being synthesized: glyoxylate, propionyl-CoA, and PA/PEP (Figure 8). Further conversion of these metabolites into TCA cycle components (Figure 9) is also versatile [39,40]. Based on this fact, a new classification of anaplerotic pathways was suggested [13]. Four groups of possible reactions giving essential intermediates were proposed:

Group (I) includes pathways of glyoxylate formation. It consists of part of glyoxylate cycle reactions, photorespiration and glyoxylate formation pathway from glycine formed at one stage of the reductive glycine pathway.

Group (II) includes pathways of propionyl-CoA and glyoxylate simultaneous formation: namely, part of methylaspartate cycle reactions, part of citramalate cycle reactions and the set of reactions of ethylmalonyl-CoA pathway.

Group (III) includes pathways of propionyl-CoA synthesis (some of the reactions of 3-hydroxypropionate and of 3-hydroxypropionate/4-hydroxybutyrate cycles).

Group (IV) includes pathways of PA/PEP formation. This group consists of subgroup A (consisting of two pathways of PA formation from exogenous acetate and CO_2_), subgroup B (including pathways of PA/PEP formation from the stored carbohydrates (glycogen)), and subgroup C (consisting of PEP/PA formation using CO_2_).

Subgroup A combines two pathways: the first pathway is PA formation through pyruvate oxidoreductase; the second pathway is PA formation involving reversible formate dehydrogenase and reversible formate-C-acetyltransferase.

Subgroup B includes the Entner–Doudoroff pathway and the Embden–Meyerhof–Parnas pathway; reactions of glycogen decomposition to β-d-fructose-6-phosphate and to β-d-glucose-6-phosphate.

Subgroup C includes the PA/PEP formation pathway (through serine) from glycine synthesized in the reductive glycine pathway and PEP/PA formation from the CBB cycle intermediates.

Pathways of further conversion of synthesized glyoxylate, propionyl-CoA, PA/PEP into TCA cycle components (Figure 9) also appear to be branched [13,39]:

I. Pathways of glyoxylate conversion: the conversion into malate (by two malate synthases different in stability and activators (EC: 2.3.3.9); or malyl-CoA/(*S*)-citramalyl-CoA-lyase (EC: 4.1.3.24/4.1.3.25) and (3*S*)-malyl-CoA-thioesterase (EC: 3.1.2.30)); the glycerate pathway for PEP formation from glyoxylate; and the pathways of glyoxylate conversion into OAA through the β-hydroxyaspartate cycle.
II. Pathways of propionyl-CoA conversion into succinyl-CoA or PA: the methylmalonyl-CoA pathway; the methylcitrate pathway; and the pathway of propionyl-CoA oxidation to PA via lactate.
III. Pathways of PA/PEP conversion into TCA cycle intermediates: PA/PEP carboxylating enzymes; the pathway of fumarate or (*S*)-malate formation from PA; and cis-aconitate formation from PA and acetyl-CoA.

The bacterium with all pathways represented in Figure 8 and Figure 9 is unlikely to exist. However, this combination of all known anaplerotic pathways in general could give a key for a quick and simple search for the anaplerotic pathway(s) in a new bacterium of interest. Additionally, it could provide insight into the active anaplerotic pathway in new bacterium on the basis of metabolomic analysis.

#### 3.1.1. Enzymes Participating in Group I Reactions

**Some glyoxylate cycle reactions.** This pathway includes some of the TCA cycle reactions (Figure 8, reactions 1–3, 11, 12, 13), the key enzyme of the glyoxylate cycle (ICL, EC: 4.1.3.1, Figure 8, reaction 14) and enzymes converting glyoxylate into malate (two pathways of glyoxylate conversion into TCA cycle intermediates are known), which are described below (Figure 9, reactions 1 or 2–3). Enzymes of TCA cycle and ICL are presented in Table 1. As a matter of fact, the same reaction can be performed by different enzymes (Table 1).

**Photorespiration.** This reaction sequence is shown in Figure 8 (reactions 15, 16 and 17, or 18–19, or 20). Synthesized in photorespiration, glycolate (ribulose-bisphosphate carboxylase (EC:4.1.1.39) catalyzed reaction 15 and phosphoglycolate phosphatase (EC: 3.1.3.18) catalyzed reaction 16, Table 2 and Table 3, respectively) is either released into the medium or enters one of four known phosphoglycolate salvage pathways [105]: the glycolate pathway (the canonical plant-like C2 cycle), where one PGA molecule is formed from two glycolate molecules, releasing one CO_2_ molecule [106]; the bacterial-like glycerate pathway; the malate cycle; and the oxalate decarboxylation pathway. Since the term photorespiration is ill-suited to describe the recycling of 2 phosphoglycolate in light-independent autotrophs, it was suggested to use a more general term “Rubisco-related 2-phosphoglycolate salvage”, or, for short, “phosphoglycolate salvage” [105,107,108,109]. The glycolate pathway is confirmed for PNSB [66,67,94].

In the phosphoglycolate salvage pathway reactions, glyoxylate is formed from glycolate [40] with the enzymes shown in Table 2. Several different enzymes can catalyze the formation of glyoxylate from glycolate (reactions 17, 18–19 or 20, Figure 8, Table 2): irreversible glycolate dehydrogenase or glycolate oxidoreductase, EC: 1.1.99.14 (reaction 17); irreversible (*S*)-2-hydroxy-acid oxidase (EC: 1.1.3.15, reaction 18) in combination with catalase (EC: 1.11.1.6, reaction 19) or catalase-peroxidase (EC: 1.11.1.21, reaction 19); NADH-dependent glyoxylate reductase (EC: 1.1.1.26/EC: 1.1.1.29, due to the possibility of catalyzing the conversion of D-glycerate to hydroxypyruvate); and NADPH-dependent glyoxylate reductase (EC: 1.1.1.79) due to a reversible formation of glyoxylate from glycolate (reaction 20, Figure 8).

Glyoxylate can be used for OAA pool replenishment after being converted to malate (Figure 9, reactions 1 or 2–3, in detail below). In this pathway, glyoxylate formation is followed by malate synthesis (catalyzed by malate synthase of glyoxylate cycle, Figure 9, reaction 1). This phosphoglycolate salvage pathway (named malate cycle) was demonstrated experimentally for *Cupriavidus necator* H16 (formerly *Ralstonia eutropha* H16 or *Alcaligenes eutrophus* H16) [105].

Ribulose-1,5-bisphosphate for this pathway could be provided by CBB cycle (Figure 8, reactions 21–32), glycogen decomposition to β-d-fructose-6-phosphate (Figure 8, reactions 37–38, 39 (or 40–43, or 43–44), 28–32) or Entner–Doudoroff pathway reactions (Figure 8, reactions 37, 38, 40–42 (or 43), 45–51, 23–32). This pathway does not work in PNSB during anaerobic phototrophic growth.

**Glyoxylate formation pathway from glycine formed at one stage of the reductive glycine pathway.** Glycine formed in the reductive glycine pathway could be converted into glyoxylate (Figure 8, reaction 75, 79–82, 85, Table 4) and further into the TCA cycle intermediates. This particular pathway produces glyoxylate from glycine by glycine dehydrogenase (EC: 1.4.2.1) or d-amino-acid oxidase (EC: 1.4.3.3); glycine transaminase (EC: 2.6.1.4); or alanine-glyoxylate aminotransferase (EC: 2.6.1.44)). This was not shown in purple bacteria. However, according to the complete genome sequencing data [110], there is a gene of the enzyme catalyzing conversion of glycine to glyoxylate in *Rba. capsulatus* (CDS, RCAP_rcc03109, alanine-glyoxylate transaminase/serine-glyoxylate transaminase/serine-pyruvate transaminase (EC: 2.6.1.44 2.6.1.45 2.6.1.51)).

#### 3.1.2. Enzymes Participating in Group II Reactions

This set of reactions lead to the formation of 2-methylfumaryl-CoA, which is converted into glyoxylate and propionyl-CoA via two reactions common for these pathways (Figure 8 and Table 5, reaction 67 and 68). Conversion of 2-methylfumaryl-CoA (mesaconyl-CoA) into L-*erythro*-3-methylmalyl-CoA (reaction 67) is catalyzed by 2-methylfumaryl-CoA hydratase (EC: 4.2.1.148). Then, L-*erythro*-3-methylmalyl-CoA is split into glyoxylate and propionyl-CoA by L-malyl-CoA lyase (EC: 4.1.3.24) or L-malyl-CoA/(*S*)-citramalyl-CoA lyase (EC: 4.1.3.24/4.1.3.25) (Figure 8, reaction 68). The subsequent transformation of synthesized propionyl-CoA and glyoxylate into the TCA cycle intermediates could occur in several reactions described below. It should be noted that even in the absence of active pathways of propionyl-CoA conversion into the TCA cycle intermediates, ethylmalonyl-CoA pathway could have an impact on TCA pool replenishment via glyoxylate emerging simultaneously with propionyl-CoA. In this case, propionyl-CoA could be used for biosynthetic needs, since it is the main participant in odd-chain fatty acid formation during growth on acetate.

**The set of reactions of ethylmalonyl-CoA pathway**, in which glyoxylate and propionyl-CoA are formed from two acetyl-CoA molecules (Figure 8, reactions 60–68). Table 5 presents information on enzymes capable of catalyzing these reactions.

The main steps of the ethylmalonyl-CoA pathway are described in Part 1. Importantly, the substrate for crotonyl-CoA synthesis is provided by acetoacetyl-CoA reductase (reaction 61a). However, crotonyl-CoA formation could be performed by other enzymes possessing substrate specificity to *S*-enanthiomers via the formation of (*S*)-3-hydroxybutanoyl-CoA from acetoacetyl-CoA (Figure 8, reaction 61b) by hydroxybutyryl-CoA dehydrogenase (EC: 1.1.1.157) or (*S*)-3-hydroxyacyl-CoA dehydrogenase (EC: 1.1.1.35), followed by crotonyl-CoA formation (Figure 8, reaction 62b) by (3*S*)-3-hydroxyacyl-CoA hydratase (EC: 4.2.1.17). Moreover, 3-hydroxyacyl-CoA dehydrogenase/enoyl-CoA hydratase/3-hydroxybutyryl-CoA epimerase (EC: 1.1.1.35; EC: 4.2.1.17; EC: 5.1.2.3), an enzyme that commonly catalyzes a reversible conversion of (*S*)-3-hydroxybutanoyl-CoA to (*R*)-3-hydroxybutanoyl-CoA (Figure 8, reaction 62c), may allow the ethylmalonyl-CoA pathway to operate as well. It also regulates the substrate flows between this pathway and ensures polyhydroxybutyrate synthesis. The (*R*)-3-hydroxybutanoyl-CoA is the substrate for the polyhydroxyalkanoate synthase (EC: 2.3.1.-, Figure 8, reaction 61c) which is *R*-specific to the isoform of the substrate.

The formation of (2*S*)-ethylmalonyl-CoA from crotonyl-CoA (reaction 63, Table 5) can catalyze crotonyl-CoA carboxylase/reductase (EC: 1.3.1.85). Reactions 64 and 65 catalyze the enzymes methylmalonyl-CoA epimerase (EC: 5.1.99.1) and ethylmalonyl-CoA mutase (EC: 5.4.99.63), respectively.

**Methylaspartate cycle reactions.** Some of the TCA cycle reactions (Figure 8, reaction 1–5, Table 1), together with some reactions of the methylaspartate cycle (Figure 8, reactions 58, 59, 56, Table 6), as well as the reactions similar to the ethylmalonyl-CoA pathway and the citramalate cycle (reactions 67 and 68, Table 5), lead to propionyl-CoA and glyoxylate formation.

Reaction 57 could be catalyzed with glutamate dehydrogenase (NADH-dependent (EC 1.4.1.2) and NADPH-dependent (EC 1.4.1.3 and EC 1.4.1.4) glutamate dehydrogenases exist). Reaction 58 is catalyzed by glutamate mutase (EC: 5.4.99.1; Figure 9, reaction 58), and reaction 59 is catalyzed by methylaspartate ammonia lyase (EC: 4.3.1.2; Figure 9, reaction 59). The reaction of coenzyme A transfer from succinyl-CoA to 2-methylfumarate giving 2-methylfumaryl-CoA (Figure 9, reaction 56) performed by succinyl-CoA:mesaconate CoA-transferase. The particular gene product is described in [112].

**Citramalate cycle reactions.** This reaction sequence is shown in Figure 8 (reactions 52–56, Table 6; 67–68, Table 5). One of two molecules (propionyl-CoA or glyoxylate) should turn into PA to replenish the first substrate of the cycle (Figure 9). This cycle is not present in the KEGG PATHWAY database in explicit form, but the sequence is shown on a separate chemical reaction level [9,10].

The first reaction of this pathway is citramalate production from PA and acetyl-CoA (reaction 53, Table 6). It can proceed directly involving (*R*)-citramalate synthase (EC: 2.3.1.182), catalyzing acetyl-CoA and PA condensation to (*R*)-citramalate with a release of coenzyme A.

Other reaction sequences resulting in citramalate formation from acetyl-CoA and PA are possible as well. The first indirect pathway of citramalate production begins (Figure 8, reactions 53a, 54a, Table 6) from (*R*)-citramalyl-CoA formation by reversible (*R*)-citramalyl-CoA lyase (EC: 4.1.3.46) followed by cleavage by succinyl-CoA:(*R*)-malate/(*R*)-citramalate CoA transferase (EC: 2.8.3.20), giving (*R*)-citramalate. The second indirect pathway of citramalate synthesis consists of two reactions (reactions 53b, 54b, Table 6). First, a reversible (*S*)-citramalyl-CoA lyase (EC: 4.1.3.25) synthesizes (*S*)-citramalyl-CoA, which is cleaved further to (*S*)-citramalate by (*S*)-citramalate-CoA transferase (EC: 2.8.3.11) or succinyl-CoA:(*S*)-malate/(*S*)-citramalate CoA-transferase (EC: 2.8.3.22). The authors of the citramalate cycle [9,10] did not specify which citramalate enantiomer is involved in its reactions. Nowadays, (*R*)-citramalate is known to be a substrate of the alternative threonine-independent isoleucine synthesis pathway [113,114] or its modification (the methylbutanoyl-CoA pathway), also containing pyruvate synthase but leading to the formation of propionyl-CoA as the final product [59]. Whereas (*S*)-citramalate is a substrate of the mesaconate pathway [115], the contribution of (*S*)-citramalyl-CoA to reactions of the 3-hydroxypropionate cycle has also been shown [18]. In addition, it was established that the enzymes catalyzing further conversion of citramalate to 2-methylfumarate (Figure 8, reaction 55) use (*S*)-enanthiomer as a substrate [116,117]. Therefore, further description of possible citramalate cycle enzymes concerns only the reactions leading to (*S*)-citramalate formation.

Subsequent synthesis of 2-methylfumarate from citramalate (reaction 55, Table 6) could be facilitated by mesaconase (EC: 4.2.1.34), which is capable of catalyzing forward and reverse 2-methylfumarate hydratation to (*S*)-citramalate. Alternatively reversible class I fumarases (EC: 4.2.1.2) could perform this reaction, since their properties described in the literature allow it [116]. Succinyl-CoA:mesaconate CoA-transferase converts 2-methylfumarate into 2-methylfumaryl-CoA (Figure 8, reaction 56, Table 6), as shown for the methylaspartate cycle [112].

Propionyl-CoA and glyoxylate are formed from 2-methylfumaryl-CoA in reactions common for the ethylmalonyl-CoA pathway and the methylaspartate cycle (Figure 9, Table 5, reactions 67, 68). The conversion reactions of propionyl-CoA and glyoxylate to the intermediates of the TCA cycle or PA are discussed in the corresponding sections below. The PA consumed in the first reaction of the citramalate cycle is replenished by decarboxylation of oxaloacetate or malate (reaction 52a, 52b or 52c, Table 6).

#### 3.1.3. Enzymes Participating in Group III Reactions

Group III comprises pathways leading to the formation of propionyl-CoA. This group is represented by one pathway including some reactions common for 3-hydroxypropionate [18] and 3-hydroxypropionate/4-hydroxybutyrate [21] cycles (Figure 6 and 7, respectively). During acetate photoassimilation, OAA pool replenishment could be achieved via the formation of propionyl-CoA Figure 8, (reactions 69, 70 or 70a–70b, 71–74), which is further converted into OAA or its precursors (Table 7).

Multisubunit acetyl-CoA-carboxylase/carboxyltransferase (EC: 6.4.1.2) carboxylates acetyl-CoA to malonyl-CoA (reaction 69). Malonyl-CoA reductase (EC: 1.1.1.75, reaction 70) produces malonate semialdehyde from malonyl-CoA. Malonate semialdehyde synthesis could be realized via another pathway (Figure 8, reactions 70a–70b [39]). Malonate is produced from malonyl-CoA by malonate CoA-transferase (EC: 2.8.3.3) or by malonyl-CoA carboxylase (EC: 4.1.1.9), which was shown to function as malonate CoA-transferase (EC: 2.8.3.3) in *Pseudomonas ovalis* [118]. Then, malonate is converted into malonate-semialdehyde (reaction 70b) by malonate-semialdehyde dehydrogenase (EC: 1.2.1.15; the amino acid sequence of this enzyme has not been determined yet [119]).

Formation of 3-hydroxypropionate from malonate-semialdehyde (reaction 71) requires 3-hydroxypropionate reductase (EC: 1.1.1.298). Reaction 71 could also be realized by 3-hydroxypropionate dehydrogenase (EC: 1.1.1.59) or malonic acid semialdehyde reductase (EC: 1.1.1.-, Table 7).

Conversion of 3-hydroxypropionate to 3-hydroxypropionyl-CoA (reaction 72) could be catalyzed by 3-hydroxypropionyl-CoA synthase (EC: 6.2.1.36), 3-hydroxybutyryl-CoA hydrolase (EC: 3.1.2.4.) or enoyl-CoA hydratase (EC 4.2.1.17, this enzyme can catalase other reactions as well). Reaction 73, conversion of 3-hydroxypropionyl-CoA to acryloyl-CoA, could be catalyzed by enoyl-CoA hydratase (EC: 4.2.1.17) instead of 3-hydroxypropionyl-CoA dehydratase (EC: 4.2.1.116) due to its broad substrate specificity. Acryloyl-CoA could be converted to propionyl-CoA (reaction 73) by a multifunctional enzyme, medium-chain acyl-CoA dehydrogenase (EC: 1.3.99.3). According to a new classification, it should be named acyl-CoA dehydrogenase (EC: 1.3.8.7). In β-alanine metabolism pathways, this enzyme plays the role of acrylyl-CoA dehydrogenase instead of the lacking analogs (EC: 1.3.1.84, acrylyl-CoA reductase (NADPH); EC: 2.8.3.12, glutaconate CoA-transferase; EC: 1.3.1.95, acrylyl-CoA reductase (NADH)).

#### 3.1.4. Enzymes Participating in Group IV Reactions

Group IV includes PA/PEP formation pathways and subdivided by three subgroups by substrates for PA/PEP synthesize.

**Subgroup A** consists of two pathways of PA formation from exogenous acetate and CO_2_ (Figure 8, reactions 75–76 or 77).

*In the first pathway*, the condensation of acetyl-CoA and CO_2_ by reversible pyruvate:ferredoxin oxidoreductase (EC: 1.2.7.1, Table 4, reaction 77) leads to PA formation. This enzyme has been studied in detail in *Rba. capsulatus* [120].

*In the second pathway* (Figure 8, reactions 75, 76, Table 4), PA could be synthesized from acetyl-CoA and formate, which is derived from CO_2_ by reversible formate dehydrogenase (EC: 1.2.1.2–EC 1.17.1.9 created in 1961 as EC 1.2.1.2, transferred in 2017 to EC 1.17.1.9) or formate:NADP^+^ oxidoreductase (formate dehydrogenase (NADP^+^), EC: 1.17.1.10). Condensation of formate and acetyl-CoA is catalyzed by reversible formate C-acetyltransferase (Pyruvate format-lyase, EC: 2.3.1.54 [121]).

The formate hydrogenlyase (FHL; formate dehydrogenase) enzyme complex is the key element of fermentative H_2_ production by *E. coli* [122], which catalyzes the disproportionation of formate to hydrogen and carbon dioxide: HCO^− 2^ + H^+^ 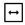 CO_2_ + H_2_. The ‘forward’ reaction (CO_2_ and H_2_ production from formate) under physiological fermentative conditions is observed [123,124]. The expression of active formate hydrogenlyase is repressed at a low formate concentration accompanied by a relatively high pH. However, theoretical arguments and experimental data indicate that this enzyme exists under certain conditions [125,126]. An evolutionary progenitor of formate hydrogenlyase on the early Earth could be responsible for a hydrogen-dependent CO_2_ fixation [127].

Pyruvate formate-lyase (PFL) is an oxygen-sensitive enzyme widespread in nature. This enzyme typically produces an additional ATP molecule (3 ATP molecules instead of two) during glucose fermentation [121]. Despite the reverse reaction of this enzyme being demonstrated in vivo, until recently its physiological function has been unknown. Zelcbuch et al. [128] demonstrated PFL-dependent co-assimilation of acetate and formate by the *E. coli* mutant strains unable to assimilate acetate through a glyoxylate shunt. In these experiments, the entire cellular pool of PA is derived from acetate and formate supporting the activity of PFL in H_2_ uptake reaction.

The oxygen-tolerant and NAD^+^-dependent formate dehydrogenase from *Rba. capsulatus* catalyzes the reduction of CO_2_ to formate [129]. The biochemical properties of this enzyme from the *Rba. capsulatus* has attracted a lot of attention [130,131].

**Subgroup B** includes pathways of PA/PEP formation from the stored carbohydrates (glycogen). The use of storage compounds is possible in periods of shift from the growth using TCA cycle intermediates to acetate as the sole organic substrate (several hours). The duration of the transition period is determined by the amount of storage compounds and the formation of the enzymes that are to be synthesized in sufficient quantity to adapt to new growth conditions. For example, the lag phase characteristic of the growth of *Rs. rubrum* with acetate with low bicarbonate concentration is also characterized by the mobilization of internal glycogen by the cell acting as a source of PA/PEP. Therefore, it may substitute the ethylmalonyl-CoA pathway as an anaplerotic pathway supporting the viability of bacteria [57]. Many bacteria use the stored carbohydrates for PEP/PA formation in *the Entner–Doudoroff pathway* (Figure 8 reactions 45–48, Table 3) along with the reactions of glycogen decomposition to β-d-glucose-6-phosphate (reactions 37, 38, 40a, 41–42 (or 43), Table 3) and in *the Embden–Meyerhof–Parnas pathway* (reactions 40, 39, 27/78, 26, 25, 24, 23, 51, 50, 49; Table 3) combined with reactions of glycogen decomposition to β-d-fructose-6-phosphate (reactions 37–38, 39 (or 40a, 41–43, or 43–44), Table 3).

**Subgroup C** includes the PA/PEP formation reaction using CO_2_. This consists of PEP/PA formation from the CBB cycle intermediates and the PA/PEP formation pathway (through serine) from glycine synthesized in reductive glycine pathway.

*PEP/PA formation from the CBB cycleintermediates.* This cycle includes reactions 21–32 shown in Figure 8 and corresponding enzymes (Table 3). The main product of CBB in heterotrophic growth conditions is 3-PGA [132]. For consequent conversion of 3-PGA into 2-PGA and then into PEP and PA, Embden–Meyerhof–Parnas pathway enzymes could be activated, namely 2,3-bisphosphoglycerate-independent phosphoglycerate mutase (EC: 5.4.2.11 or EC: 5.4.2.12), PEP-hydratase (EC: 4.2.1.11) and pyruvate kinase (EC: 2.7.1.40)—Figure 8, reactions 51, 50, 49, respectively.

During the photoheterotrophic growth using acetate, lactate, malate, and succinate, PNSB produce carbon dioxide, since the carbon atom in the biomass is more reduced than in these compounds [133]. However, this does not mean that purple bacteria do not require exogenous carbon dioxide or carbonate ions. This follows from the fact that at elevated pH and a high content of Ca and Mg ions in the medium, the produced mineral carbon can participate in reactions of precipitation. It should be noted that exogenous carbon dioxide is required at high concentrations of organic acids in the growth medium (apparently it is not connected with the redox balancing of cells) or under bacterial photoheterotrophic growth conditions at high light intensities (discussed in Part 3).

In some cases, especially when the acetate cultures grow at the maximum growth rate, the role of the cycle might be shifted to the anaplerotic pathway for the replenishment of TCA cycle intermediates. This suggestion needs further experimental verification.

*The PA/PEP formation pathway (through serine) from glycine synthesized in reductive glycine pathway* (reaction 84, 88–93 Table 4, Figure 8). An alternative hypothetical variant of the reductive glycine pathway relies on the synthesis of PA from serine [24]. In this pathway, instead of the deamination step, serine can be synthesized from glycine and 5,10-methylen-THF (product of previous glycine formation). After that, serine can be converted into PA. This sequence of reactions in combination with pyruvate carboxylase can also replenish the pool of TCA cycle intermediates.

The reversible formate dehydrogenase forms formate from CO_2_ and H_2_/H^+^ (Figure 8, reaction 75). Formate-TNF ligase, formate transporter, methenyl-TNF cyclohydrolase dehydrogenase (reaction 80, 81), glycine cleavage/synthase system (reaction 82), ammonia importer) work in concert, transforming formate into glycine (Figure 8 reaction 79). Serine hydroxymethyltransferase synthesizes serine from glycine (reaction 83). Finally, serine deaminase (reaction 84) transforms serine into PA. This pathway has not been shown for PNSB.

### 3.2. Further Conversion of Synthesized Glyoxylate, Propionyl-CoA and PA/PEP into TCA Cycle Intermediates

#### 3.2.1. Glyoxylate Conversion 

**Glyoxylate to Malate Conversion.** At present, two pathways of glyoxylate conversion into TCA cycle intermediates are known; both lead to (*S*)-malate formation.

The most well-known pathway is the condensation of glyoxylate and acetyl-CoA to (*S*)-malate involving *malate synthase* (EC: 2.3.3.9) (Figure 9, Table 8, reaction 1). Two different genes produce malate synthases (EC: 2.3.3.9): malate synthase A and malate synthase G. These enzymes differ in stability and the nature of their inhibitors. Malate synthase G is strongly inducible by glycolate (by 1000 times) and glyoxylate [134]. Malate synthase A is 20-fold induced by acetate or fatty acids [135]. Malate synthase G is related to glycolate metabolism in some bacteria [136,137].

The conversion of glyoxylate to malate could take place via the subsequent action of *malyl-CoA/(S)-citramalyl-CoA-lyase* (EC: 4.1.3.24/4.1.3.25), which catalyzes the formation of L-malyl-CoA, and *(3S)-malyl-CoA-thioesterase* (EC: 3.2.1.30), thus catalyzing the formation of (*S*)-malate from L-malyl-CoA (Figure 9, reactions 2 and 3, respectively; Table 8 [111]).

**Glycerate Pathway for PEP Formation from Glyoxylate.** The pathways of glyoxylate conversion through the reaction of the glycerate pathway were determined [138,139]. The reactions of this pathway are shown in Figure 9 (reactions 32–35, Table 8, simultaneously with pathways of PA/PEP conversion into TCA cycle intermediates (Table 9)). Two molecules of glyoxylate by glyoxylate carboligase condense into tartronate semialdehyde with CO_2_ released (reaction 32). Tartronate semialdehyde reductase catalyzes the formation of d-glycerate from tartronate semialdehyde (reaction 33). d-glycerate is converted to 2-phospho-d-glycerate under the action of the glycerate kinase (reaction 34). It is then cleaved by enolase to PEP and H_2_O (reaction 35). PEP is carboxylated to oxaloacetate phosphoenolpyruvate carboxylase (or by alternative reactions: reaction 20, or through the PA formation step is converted to TCA cycle intermediates, Figure 9; more details in the section on the carboxylation of PA/PEP are given below).

**β-hydroxyaspartate Cycle for OAA Formation from Glyoxylate.** The β-hydroxyaspartate cycle was discovered in the middle of the last century [140], and yet was only described in detail in 2019 [141]. In this cycle, one molecule of oxaloacetate is formed from two molecules of glyoxylate (Figure 9 of the reaction 28–31, 28; Table 8). The enzyme aspartate-glyoxylate aminotransferase catalyzes one reaction, which is both the first and the last reaction in the cycle (Figure 9, reaction 28): glycine (involved in subsequent reactions of the cycle) and oxaloacetate (the final product of β-hydroxyaspartate cycle) are formed from glyoxylate and aspartate. Glycine and the second molecule of glyoxylate under the action of β-hydroxyaspartate aldolase are converted to (2R, 3*S*)-β-hydroxyaspartate (Figure 9, reaction 29). From the latter, under the action of β-hydroxyaspartate dehydratase, iminosuccinate and H_2_O are formed (reaction 30). Iminosuccinate reductase catalyzes the formation of an aspartate molecule from iminosuccinate (reaction 31), which enters the first cycle reaction (reaction 28). One of its products is an oxaloacetate molecule synthesized from two glyoxylate molecules; in addition, glycine is formed as described above (one of the substrates of the second reaction of the cycle). The β-hydroxyaspartate cycle is present in the genomes of some Proteobacteria that also encode the CBB cycle [141], but the presence in PNSB needs additional investigation.

#### 3.2.2. Pathways of Propionyl-CoA Conversion into Succinyl-CoA or PA

There are three pathways of propionyl-CoA conversion into TCA cycle intermediates replenishing OAA pool known up to date. The methylmalonyl-CoA pathway leads to the formation of succinyl-CoA from propionyl-CoA [142]). In other pathways (methylcitrate pathway and oxidative pathway via lactate), propionyl-CoA is converted into PA, which could yield malate or OAA in several reaction sequences, as described in the corresponding sections.

**Methylmalonyl-CoA Pathway.** Succinyl-CoA is formed in this pathway from propionyl-CoA using three reactions (Figure 9, reactions 4–6; Table 10). (*S*)-methylmalonyl-CoA formation from propionyl-CoA (reaction 4) can be catalyzed by two enzymes, propionyl-CoA carboxylase (EC: 6.4.1.3) and methylmalonyl-CoA carboxytransferase (EC: 2.1.3.1). Methylmalonyl-CoA/ethylmalonyl-CoA epimerase (EC: 5.1.99.1) isomerizes (*S*)-methylmalonyl-CoA into (*R*)-methylmalonyl-CoA (reaction 5). Methylmalonyl-CoA mutase (EC: 5.4.99.2) catalyzes the formation of succinyl-CoA from (*R*)-methylmalonyl-CoA (reaction 6).

**Methylcitrate Cycle.** The sequence of this pathway reactions is given in Figure 9 (reactions 11, 12 (or 12a-12b), 13, 14; Table 10), where PA and succinate are produced, succinate being directed to compensate the first steps of this cycle (Figure 8, reaction 11–13). PA can be involved in TCA cycle intermediate formation reactions (see below). The pathway has two modifications [143,144] that differ in the enzymes catalyzing reaction 12. In the first methylcitrate cycle, this reaction is catalyzed by methylcitrate dehydratase (EC: 4.2.1.79), whereas in the second methylcitrate cycle 2-methyl-*cis-*aconitate formation is achieved via two reactions (Figure 9, reactions 12a and 12b), i.e., via 2-methyl-*trans*-aconitate formation by 2-methylcitrate dehydratase (2-methyl-*trans*-aconitate forming; EC: 4.2.1.117) and 2-methylaconitate isomerase (EC: 5.2.1.-).

The gene of aconitate hydratase/2-methylisocitrate hydratase (EC: 4.2.1.3/EC: 4.2.1.99) is absent in the genomes of all studied *Rps. palustris* strains and *Rba. capsulatus* [13]. However, classic aconitate hydratase (EC: 4.2.1.3, Figure 8, reactions 2–3; Figure 9, reaction 13; Table 10) was shown to play a bifunctional role in *Salmonella enterica* [145]. Such possibility for aconitate hydratase in PNSB requires experimental validation.

**Pathway of Propionyl-CoA Oxidation via Lactate to PA.** The scheme of this pathway is shown in Figure 9, reactions 7–10 (Table 10). The following enzymes could be used to catalyze acryloyl-CoA formation from propionyl-CoA (reaction 7): acrylyl-CoA reductases (NADPH) (EC: 1.3.1.84), glutaconate CoA-transferases (EC: 2.8.3.12), acrylyl-CoA reductase (NADH) (EC: 1.3.1.95) or acyl-CoA dehydrogenase, which could perform the function of acrylyl-CoA reductase in pathways of β-alanine metabolism. Reaction 8 is catalyzed by lactoyl-CoA dehydratase (EC: 4.2.1.54), reaction 9, and by propionate CoA-transferase (EC: 2.8.3.1). Lactate transformation to PA (reaction 10) could be catalyzed by lactate dehydrogenase (EC: 1.1.1.27), L-lactate dehydrogenase (cytochrome) (EC: 1.1.2.3) or malate-lactate transhydrogenase (EC: 1.1.99.7).

#### 3.2.3. Pathways of TCA Cycle Intermediate Formation from PA/PEP

**PA/PEP Carboxylating Enzymes (Anaplerotic Carboxylases).** Synthesis of the TCA cycle intermediates from PA or PEP requires their carboxylation, which could lead to (*S*)-malate or oxaloacetate formation (Figure 9, reactions 19, 19a, 20–22; Table 9 [1]). Six enzymes capable of anaplerotic carboxylation exist: PEP carboxylase (EC: 4.1.1.31; reaction 21), GTP-dependent PEP carboxykinase (EC: 4.1.1.32; reaction 20) and PEP carboxykinase (diphosphate) (EC: 4.1.1.38; reaction 20); ATP-dependent PEP carboxykinase (EC: 4.1.1.49, reaction 20); irreversible pyruvate carboxylase (EC: 6.4.1.1., reaction 22); and reversible malate dehydrogenase (oxaloacetate decarboxylating) (EC: 1.1.1.38 and EC:1.1.1.40, reactions 19, 19a or EC: 1.1.1.39, reaction 19a).

The favorable direction of the malate dehydrogenase (oxaloacetate decarboxylating) reaction is decarboxylation. However, some malic enzymes demonstrated a pH-dependence of this favorable reaction direction (to PA or malate formation) [146]. In addition to this, malic enzyme was shown to be a key enzyme in replenishing TCA cycle intermediates in *Synechocystis* sp. PCC 6803, because it catalyzes PA carboxylation [147]. Among three theoretically reversible malate dehydrogenases (oxaloacetate decarboxylating) (EC: 1.1.1.40, EC: 1.1.1.38, EC: 1.1.1.39), only one gene coding EC: 1.1.1.40 is present in all the studied *Rps. palustris* strains and *Rba. capsulatus* SB1003 [13]. However, in *Rba. capsulatus*, it is encoded by two genes.

Among the enzymes catalyzing the reversible PEP carboxylation to OAA (Figure 9, reaction 20), one PEP carboxykinase (EC: 4.1.1.49) gene is present in all studied *Rps. palustris* strains and *Rba. capsulatus* SB1003 [13]. Studies have described the contribution of this enzyme to PA and acetate metabolism [35,132]. This enzyme was shown to be able to catalyze the reverse reaction, i.e., C3-carboxylation, for example, in *Rl. eutropha* [148,149].

Several *Rps. palustris* strains have a gene of PEP carboxylase enzyme catalyzing irreversible PEP carboxylation to oxaloacetate (Figure 9, reaction 21), *Rba. capsulatus* SB1003 lacks this gene [13].

**Fumarate Formation from PA and CO_2_.** In this pathway (Figure 9, reactions 23–25; Table 9), malic enzyme-dependent carboxylation of PA to (*R*)-malate can occur (EC: 1.1.1.83), but (*R*)-malate cannot be directly metabolized in the TCA cycle. (*R*)-malate is converted into maleate by maleate hydratase (reaction 24, EC: 4.2.1.31). Maleate can be further converted into fumarate by maleate isomerase (reaction 25, EC: 5.2.1.1). The latter is involved into TCA cycle reactions.

A multifunctional enzyme discovered in *Methanocaldococcus jannaschii* encoded by *leuC* gene [144] is currently associated with maleate hydratase function (EC: 4.2.1.31). It was shown to display maleate hydratase activity [150].

Transformation of (*R*)-malate to (*S*)-malate could be provided by enzyme having racemase activity (Figure 9, reaction 26; Table 9). The possibility of this reaction was demonstrated experimentally in *Rba. capsulatus* cultures [151]. Unfortunately, information on the coding gene (genes), properties and structure of this enzyme is not available.

***Cis*-Aconitate Formation from PA and Acetyl-CoA.** A reaction sequence related to the itaconate metabolism (Figure 9, reactions 15–17, 18 (or 18a, 18b); Table 9) could hypothetically take part in TCA cycle intermediate replenishment on the *cis*-aconitate level [40]. This path is considered to be the main means of assimilating itaconate in bacteria [152]. The itaconic metabolism acid has been elucidated in the ascomycetous fungus *Aspergillus terreus* [153], in the basidiomycetous fungus *Ustilago maydis* [154] via *trans*-aconitate, and in human macrophages via *cis*-aconitate. All these reactions, except for *cis*-aconitate decarboxylation to itaconate (reaction 18 or 18b), are considered to be reversible nowadays. As mentioned above, some carboxylyases (decarboxylases) direct the reaction depending on the environmental conditions (substrate/product concentrations, pH, etc.) [146,147]. The reversion of reaction 18 (or 18a) could lead to the formation of aconitate from acetate and PA. There are no sufficient data on the possibility of converting itaconate in reactions 18a and 18b accounted for scarce information on itaconate-converting enzymes. As of today, only a few enzymes from quite a low number of organisms have been characterized [155]. However, there are a few experimental studies into enzymatic activities of itaconate converting reactions.

All the reactions listed above are unique for the putative *cis*-aconitate formation pathway except for the condensation of PA and acetyl-CoA (Figure 9, reaction 53 or Figure 9, reaction 15; Table 9). The latter reaction is catalyzed by (*S*)-citramalyl-CoA lyase (EC: 4.1.3.25), and itaconyl-CoA conversion to itaconate is performed by succinyl-CoA synthetase (EC: 6.2.1.4, 6.2.1.5, reaction 17, Figure 9).

Conversion of (3*S*)-citramalyl-CoA into itaconyl-CoA (Figure 9, reaction 16) is catalyzed by itaconyl-CoA hydratase (EC: 4.2.1.56). Then, itaconyl-CoA is converted into itaconate (reaction 17) by succinyl-CoA:itaconate CoA-transferase (EC: 2.8.3.-) or succinyl-CoA synthetase (EC: 6.2.1.4, 6.2.1.5). *Cis*-aconitate decarboxylase (EC: 4.1.1.6, reaction 18) further carboxylates itaconate to *cis*-aconitate.

## 4. Part 3: Alternative Functions of Anaplerotic Pathways

Evidently, the replenishment of the TCA cycle intermediates is vital for any microorganism, and purple bacteria are no exception. Nevertheless, the carbon metabolism in purple bacteria, as in other prokaryotes, is highly complex and flexible. That is why the anaplerotic pathways, in addition to TCA intermediate pool replenishment, could play different alternative roles. Most of them are also vital for the bacterium’s survival, and improve its ability to withstand stress or compete with other species in the environment.

For example, an elevated concentration of acetate prevents the growth of *E. coli* and other bacteria [156]. There are several reports on the inhibiting effect of elevated acetate concentration for purple bacteria such as *Rba. capsulatus* [38,157] and *Rs. rubrum* [57]. This property of acetate is widely used in the food industry, which applies acetic acid as a preservative. Several explanations have been put forward to account for the reasons why a high acetate concentration inhibits microorganism growth. Among them are difficulty of the membrane’s potential maintenance [158], increased intracellular osmotic pressure [159,160], and a disruption of carbon flows in central metabolism, which itself inhibits growth [161,162,163]. None of these hypotheses have been proven. The disruption of acetyl-phosphate showed some growth-inhibiting effects (~20%), and uncoupling the inhibiting effect plays only a limited role [156]. In all cases, anaplerotic pathways actively contribute to acetate consumption/degradation, with a decreased inhibitory effect of high acetate concentrations on bacterial growth [1,57].

Sustainable functioning of CO_2_-consuming anaplerotic pathways under a high acetate concentration can be supported by adding bicarbonate or carbon dioxide as an additional electron acceptor [3,15,164]). For example, the increase in acetate concentration from 1 to 100 mM increases the lag period and reduces the growth rate of *Rba. capsulatus* strain St. Luis [38]. Another strain of this bacterium could not grow at a high concentration of acetate without bicarbonate addition [165]. The CBB cycle and other pathways of carbon dioxide assimilation in this case work as electron sink spending excess of electrons but not as anaplerotic pathway [15,51]. Another way of maintaining the redox balance for purple bacteria grown with acetate is not connected with anaplerotic pathways but with nitrogen fixation. In this process, the nitrogenase system acts as an electron sink using electrons for nitrogen fixation and hydrogen production. The regulatory system in organisms regulates both CBB and nitrogen fixation processes [83]. This system is different in various types of purple bacteria (see above, PART 1, The CBB cycle), but the main goal of them is redox balance maintenance inside cells.

Excessive illumination is a stress for purple bacteria grown with acetate. An increase in the light intensity results in an excess of the proton motive force. This brings about an excessive NADH synthesis via a reverted electron flow by NADH dehydrogenase [166,167]. A decrease in illumination or additional bicarbonate restored the growth of bacteria [57].

Anaplerotic pathways acceleration could eliminate strong light growth inhibition. For example, *Rs. rubrum* S1H after a prolonged cultivation with acetate produced an “acetate-competent” strain [51]. It appears that this strain contained several additional copies of ethylmalonyl-CoA pathway genes due to a reversible gene duplication and amplification. This particular strain did not have any lag-phase at the growth onset with acetate and tolerated strong light.

Changes in the synthesis of storage compounds are yet another mechanism to adapt to the stress of elevated light and acetate assimilation [168,169]. Purple bacteria are capable of accumulating polyhydroxyalkanoates (PHA) [170,171]. PHA is the major storage compound in bacteria grown on acetate. A protein profile with analysis of the PHA content in *Rs. rubrum* wild type and “acetate-competent” strains showed that the “acetate-competent” strain could resist strong light much better than a wild strain [57]. During the switch to strong light, the “acetate-competent” strain was demonstrated to grow better and did not accumulate the elevated quantity of PHA, in contrast to the wild strain. Thus, the wild strain involved PHA synthesis in light-stress adaptation, whereas “acetate-competent strain” did not due to the higher activity of PHA synthesis.

Thus, the genetically determined anaplerotic pathways may take part in various critical processes underlying the adaptation of purple bacteria to environmental stresses.

## 5. Conclusions

The data reviewed in this paper reveal that our understanding of anaplerotic pathways replenishing the TCA cycle intermediates in microorganisms and, particularly, in purple bacteria has expanded significantly in the last decade. This success results from advances in genomic sequencing and proteomic, transcriptomic, and metabolomic research. We have already known that the simultaneous functioning of more than one pathway is not an exception but rather a rule for numerous microorganisms [172]. The integrated network of all known anaplerotic pathways in one scheme has appeared recently [13,39]. This shows that central carbon metabolism is very flexible. Simultaneously, this network shows that all known anaplerotic pathways share many common reaction chains. This integrated scheme might efficiently outline active anaplerotic pathways in a newly discovered strain. Nowadays, the quickest way to elucidate it is the genome sequencing followed by the analysis of genetic potential for different anaplerotic pathways. After this initial step, the exact transcriptomic experiments with subsequent proteomic analysis provide clues for the final metabolomics study.

Our overview of possible anaplerotic pathways show that many reaction chains/cycles able to replenish TCA cycle intermediates have some additional functions. Furthermore, some chains were described previously as having another function (for example, CBB cycle). Thus, central carbon metabolism is a sophisticated system supporting the lives of microbials life, and our classification of different chains for anaplerotic function, inorganic carbon acquisition, synthesis of different blocks for overall metabolism or energetic reactions is just our simplification of their nature.

## Figures and Tables

**Table 1:** Pathways of glyoxylate formation including some glyoxylate cycle reactions (reactions 1–3, 14, 11–13) and TCA cycle enzymes (Figure 8, reactions 1–13).

Reaction Number in Figure 8	Function Number, Enzyme Name and Number (KEGG Orthology Database)
1	K01647 citrate synthase [EC:2.3.3.1]
2, 3	K01681 aconitate hydratase [EC:4.2.1.3]
4, 5	K00031 isocitrate dehydrogenase [EC:1.1.1.42]
4, 5/1	K00906 isocitrate dehydrogenase kinase/phosphatase [EC:2.7.11.5 3.1.3.-]
4a	K00030 isocitrate dehydrogenase (NAD+) [EC:1.1.1.41]
4a	K17753 isocitrate—homoisocitrate dehydrogenase [EC:1.1.1.286]
6, 7	K00164 2-oxoglutarate dehydrogenase E1 component [EC:1.2.4.2]
8	K00658 2-oxoglutarate dehydrogenase E2 component (dihydrolipoamide succinyltransferase) [EC:2.3.1.61]
9	K00382 dihydrolipoamide dehydrogenase [EC:1.8.1.4]
6a	K00174 2-oxoglutarate/2-oxoacid ferredoxin oxidoreductase subunit alpha [EC:1.2.7.3 1.2.7.11]
6b	K00175 2-oxoglutarate/2-oxoacid ferredoxin oxidoreductase subunit beta [EC:1.2.7.3 1.2.7.11]
10	K01902 succinyl-CoA synthetase alpha subunit [EC:6.2.1.5]
	K01903 succinyl-CoA synthetase beta subunit [EC:6.2.1.5]
10	K18118 succinyl-CoA:acetate CoA-transferase [EC:2.8.3.18]
10	K01899 succinyl-CoA synthetase alpha subunit [EC:6.2.1.4 6.2.1.5]
	K01900 succinyl-CoA synthetase beta subunit [EC:6.2.1.4 6.2.1.5]
11	K00239 succinate dehydrogenase/fumarate reductase, flavoprotein subunit [EC:1.3.5.1 1.3.5.4]
	K00240 succinate dehydrogenase/fumarate reductase, iron-sulfur subunit [EC:1.3.5.1 1.3.5.4]
	K00242 succinate dehydrogenase/fumarate reductase, membrane anchor subunit
	K00241 succinate dehydrogenase/fumarate reductase, cytochrome b subunit
12	K01679 fumarate hydratase, class II [EC:4.2.1.2]
12	K01676 fumarate hydratase, class I [EC:4.2.1.2]
13 *	K00024 malate dehydrogenase [EC:1.1.1.37]
13 *	K00116 malate dehydrogenase (quinone) [EC:1.1.5.4]
14	K01637 isocitrate lyase [EC:4.1.3.1]

Footnote: *—It was shown that the tetrameric form of malate dehydrogenase of *Rps. palustris* strain f-8pt enables the glyoxylate cycle and the dimeric form provides for TCA cycle to operate [104].

**Table 2:** The enzymes of glyoxylate formation involving Rubisco oxygenase activity (photorespiration) (Figure 8, reactions 15, 16 and 17, or 18–19, or 20). Information on reaction 15 (Table 3) is given below.

Reaction Number in Figure 8	Function Number, Enzyme Name and Number (KEGG Orthology Database)
16	K01091 phosphoglycolate phosphatase [EC:3.1.3.18]
17	No function number in KEGG; glycolate dehydrogenase [1.1.99.14]
18	K11473 glycolate oxidase iron-sulfur subunit
	K11472 glycolate oxidase FAD binding subunit
	K00104 glycolate oxidase [EC:1.1.3.15]
19	K03782 catalase-peroxidase [EC:1.11.1.21]
19	K03781 catalase [EC:1.11.1.6]
20	K00015 glyoxylate reductase [EC:1.1.1.26]
20	K12972 glyoxylate/hydroxypyruvate reductase [EC:1.1.1.79; 1.1.1.81]

**Table 3:** PA/PEP formation pathway from the CBB cycle intermediates (21–32, 51, 50, 49); decomposition of the glycogen reserved earlier to ribulose-1,5-bisphosphate with participation of reactable 6. phosphate (reactions 37, 38, 39 (or 40a–41–43, or 43–44), 28–32); the Embden–Meyerhof–Parnas pathway (reactions 38, 39, 27/78, 26, 25, 24, 23, 51, 50, 49); the Entner–Doudoroff pathway (45–48) and reactions 37, 38, 40a-41–42 (or 43), 45–51, 23–32. The reaction of glycogen degradation (37) and biosynthesis (33, 33a, 34, 35, 36).

Reaction Number in Figure 8	Function Number, Enzyme Name and Number (KEGG Orthology Database)
21	K00855 phosphoribulokinase [EC: 2.7.1.19]
15, 22	K01601 ribulose-bisphosphate carboxylase large chain [EC: 4.1.1.39]
	K01602 ribulose-bisphosphate carboxylase small chain [EC: 4.1.1.39]
23	K00927 phosphoglycerate kinase [EC: 2.7.2.3]
24	K00134 glyceraldehyde 3-phosphate dehydrogenase [EC: 1.2.1.12]
25	K01803 triosephosphate isomerase (TIM) [EC: 5.3.1.1]
26, 29	K01623 fructose-bisphosphate aldolase, class I [EC: 4.1.2.13]
26,29	K01624 fructose-bisphosphate aldolase, class II [EC: 4.1.2.13]
27	K03841 fructose-1,6-bisphosphatase I [EC: 3.1.3.11]
27, 30	K11532 fructose-1,6-bisphosphatase II/sedoheptulose-1,7-bisphosphatase [EC: 3.1.3.11 3.1.3.37]
28, 31	K00615 transketolase [EC: 2.2.1.1]
32	K01807 ribose 5-phosphate isomerase A [EC: 5.3.1.6]
32	K01808 ribose 5-phosphate isomerase B [EC: 5.3.1.6]
33	K00975 glucose-1-phosphate adenylyltransferase [EC: 2.7.7.27]
33a	K00963 UTP—glucose-1-phosphate uridylyltransferase [EC: 2.7.7.9]
34	K00693/K16150 glycogen synthase [EC: 2.4.1.11]
34	K00750 glycogenin [EC: 2.4.1.186]
34	K16153 glycogen phosphorylase/synthase [EC: 2.4.1.1 2.4.1.11]
34	K13679/K20812 granule-bound starch synthase [EC: 2.4.1.242]
35	K00703 starch synthase [EC: 2.4.1.21]
35	K13679 granule-bound starch synthase [EC: 2.4.1.242]
35	K20812/K13679 glycogen synthase [EC: 2.4.1.242]
36	K00700 1,4-alpha-glucan branching enzyme [EC: 2.4.1.18
37	K00688 glycogen phosphorylase [EC: 2.4.1.1]
37	K16153 glycogen phosphorylase/synthase [EC: 2.4.1.1 2.4.1.11]
37	K01196 glycogen debranching enzyme [EC: 2.4.1.25 3.2.1.33]
37	K02438 glycogen debranching enzyme [EC: 3.2.1.196]
37	K01200 pullulanase [EC: 3.2.1.41]
38	K01835 phosphoglucomutase [EC: 5.4.2.2]
38	K15779 phosphoglucomutase/phosphopentomutase [EC: 5.4.2.2 5.4.2.7]
38	K15778 phosphomannomutase/phosphoglucomutase [EC: 5.4.2.8 5.4.2.2]
39, 43, 44	K01810 glucose-6-phosphate isomerase [EC: 5.3.1.9]
39	K00688 glycogen phosphorylase [EC: 2.4.1.1]
39	K16153 glycogen phosphorylase/synthase [EC: 2.4.1.1 2.4.1.11]
39	K01196 glycogen debranching enzyme [EC: 2.4.1.25 3.2.1.33]
40, 42	K00845 glucokinase [EC: 2.7.1.2]
40, 42	K12407 glucokinase [EC: 2.7.1.2]
40, 42	K00844 hexokinase [EC: 2.7.1.1]
40a	K01084 glucose-6-phosphatase [EC: 3.1.3.9]
41	K01785 aldose 1-epimerase [EC: 5.1.3.3]
45	K00036 glucose-6-phosphate 1-dehydrogenase [EC: 1.1.1.49 1.1.1.363]
45	K19243 NAD+ dependent glucose-6-phosphate dehydrogenase [EC: 1.1.1.388]
46	K01057, K07404 6-phosphogluconolactonase [EC: 3.1.1.31]
47	K01690 phosphogluconate dehydratase [EC: 4.2.1.12]
48	K01625 2-dehydro-3-deoxyphosphogluconate aldolase/(4*S*)-4-hydroxy-2-oxoglutarate aldolase [EC: 4.1.2.14 4.1.3.42]
49	K00873 pyruvate kinase [EC: 2.7.1.40]
50	K01689 enolase [EC: 4.2.1.11]
51	K15633 2,3-bisphosphoglycerate-independent phosphoglycerate mutase [EC: 5.4.2.12]
51	K01834 2,3-bisphosphoglycerate-dependent phosphoglycerate mutase [EC: 5.4.2.11]
78	K16370 6-phosphofructokinase 2 [EC: 2.7.1.11]

**Table 4:** Glyoxylate formation pathway from glycine synthesized in reductive glycine pathway (reactions 75, 79–82, 85). The enzymes taking part in PA formation from exogenous acetate (reactions 75–76 or 77) and PA/PEP formation pathway (through serine) from glycine synthesized in the reductive glycine pathway (reaction 75, 79–84).

Reaction Number in Figure 8	Function Number, Enzyme Name and Number (KEGG Orthology Database)
75	K15022 formate dehydrogenase (NADP+) beta subunit [EC: 1.17.1.10]
75	K00126 formate dehydrogenase subunit delta [EC: 1.17.1.9]
	K00123 formate dehydrogenase major subunit [EC: 1.17.1.9]
	K00124 formate dehydrogenase iron-sulfur subunit
	K00127 formate dehydrogenase subunit gamma
76	K00656 formate C-acetyltransferase [EC: 2.3.1.54]
77	K03737 pyruvate-ferredoxin/flavodoxin oxidoreductase [EC: 1.2.7.1 1.2.7.-]
-	K06212 formate transporter
79	K01938 formate—tetrahydrofolate ligase [EC 6.3.4.3]
80	K01500 methenyltetrahydrofolate cyclohydrolase [EC: 3.5.4.9]
80,81	K01491 methylenetetrahydrofolate dehydrogenase (NADP+)/methenyltetrahydrofolate cyclohydrolase [EC: 1.5.1.5 3.5.4.9]
81	K00300 methylenetetrahydrofolate/methylenetetrahydromethanopterin dehydrogenase (NADP+) [EC: 1.5.1.5 1.5.1.-]
-	No function number in KEGG; ammonia importer
82a	K00605 aminomethyltransferase [2.1.2.10]
82b	K00282 glycine dehydrogenase subunit 1 [EC: 1.4.4.2]
82b	K00283 glycine dehydrogenase subunit 2 [EC: 1.4.4.2]
82c	K00382 dihydrolipoamide dehydrogenase [1.8.1.4]
83	K00600 glycine hydroxymethyltransferase [2.1.2.1]
84	K17989 L-serine/L-threonine ammonia-lyase [4.3.1.19]
85	No function number in KEGG; glycine dehydrogenase (cytochrome); (EC: 1.4.2.1)

**Table 5:** Pathways of propionyl-CoA and glyoxylate simultaneous formation: the set of ethylmalonyl-CoA pathway enzymes (reactions 60, (61a, 62a)/61b, 62b), 63–68).

Reaction Number in Figure 8	Function Number, Enzyme Name and Number (KEGG Orthology Database)
60	K00626 acetyl-CoA C-acetyltransferase [EC: 2.3.1.9]
61a	K00023 acetoacetyl-CoA reductase [EC: 1.1.1.36]
62a	K17865 3-hydroxybutyryl-CoA dehydratase [EC: 4.2.1.55]
61b/62b	K01782 3-hydroxyacyl-CoA dehydrogenase/enoyl-CoA hydratase/3-hydroxybutyryl-CoA epimerase [EC: 1.1.1.35 4.2.1.17 5.1.2.3]
61b/62b	No function number in KEGG; fatty acid oxidation complex, α-subunit (EC: 1.1.1.35; 4.2.1.17; 5.3.3.8)
61b	No function number in KEGG; 3-hydroxyacyl-CoA dehydrogenase/3-hydroxy-2-methylbutyryl-CoA dehydrogenase (EC: 1.1.1.178; 1.1.1.35)
61b	K00074 3-hydroxybutyryl-CoA dehydrogenase [EC: 1.1.1.157]
62b	K01715 enoyl-CoA hydratase [EC: 4.2.1.17]
61c	K03821 polyhydroxyalkanoate synthase subunit PhaC [EC: 2.3.1.-]
62c	K01782 3-hydroxyacyl-CoA dehydrogenase/enoyl-CoA hydratase/3-hydroxybutyryl-CoA epimerase [EC: 1.1.1.35 4.2.1.17 5.1.2.3]
62c	K01825 3-hydroxyacyl-CoA dehydrogenase/enoyl-CoA hydratase/3-hydroxybutyryl-CoA epimerase/enoyl-CoA isomerase [EC: 1.1.1.35 4.2.1.17 5.1.2.3 5.3.3.8]
63	K14446 crotonyl-CoA carboxylase/reductase [EC: 1.3.1.85]
64	K05606 methylmalonyl-CoA/ethylmalonyl-CoA epimerase [EC: 5.1.99.1]
65	K14447 ethylmalonyl-CoA mutase [EC: 5.4.99.63]
66	K14448 (2*S*)-methylsuccinyl-CoA dehydrogenase [EC: 1.3.8.12]
67	K14449 2-methylfumaryl-CoA hydratase [EC: 4.2.1.148]
68,53 (Figure 8) and 2 (Figure 9)	K08691 malyl-CoA/(*S*)-citramalyl-CoA lyase [EC: 4.1.3.24 4.1.3.25] *
3 (Figure 9)	K14451 (3*S*)-malyl-CoA thioesterase [EC: 3.1.2.30] *

Footnote: *—citrate lyase β- subunits 1 and 2 are paralogs of one of citrate lyase components, EC: 4.1.3.22, from *Clostridium tetanomorphum*. An ability of citrate lyase β-subunit 1 to function as (3*S*)-malyl-CoA-thioesterase (EC: 3.1.2.30), and that of citrate lyase β-subunit 2 to function as malyl-CoA-liase/(*S*)-citramalyl-CoA lyase (EC: 4.1.3.24: 4.1.3.25) in *Rba. sphaeroides* was shown experimentally [111].

**Table 6:** Pathways of propionyl-CoA and glyoxylate simultaneous formation: the part of methylaspartate cycle reactions (reactions 57–59, 56, 67–68 *), the part of citramalate cycle (reactions 52, 53b, 54b, 55, 56, 67–68 *) and reactions of (*R*)-citramalate synthesis (53 or 53a–54a).

Reaction Number in Figure 8	Function Number, Enzyme Name and Number (KEGG Orthology Database)
57	K00260 glutamate dehydrogenase [EC: 1.4.1.2]
57	K00261 glutamate dehydrogenase (NAD(P)+) [EC: 1.4.1.3]
57	K15371 glutamate dehydrogenase [EC: 1.4.1.2]
58	K19268 methylaspartate mutase epsilon subunit [EC: 5.4.99.1]
59	K04835 methylaspartate ammonia-lyase [EC: 4.3.1.2]
56	K19280 succinyl-CoA:mesaconate CoA transferase [EC: 2.8.3.26]
53	K09011 (*R*)-citramalate synthase [EC: 2.3.1.182]
53a	K18314 (*R*)-citramalyl-CoA lyase [EC:4.1.3.46]
54a	K18313 succinyl-CoA—D-citramalate CoA-transferase [EC: 2.8.3.20]
53b	K18292 (*S*)-citramalyl-CoA lyase [EC: 4.1.3.25]
54b	No function number in KEGG; (*S*)-citramalate-CoA transferase (EC: 2.8.3.11)
54b	No function number in KEGG; succinyl-CoA:(*S*)-malate/(*S*)-citramalate CoA-transferase (EC: 2.8.3.22)
55	No function number in KEGG; mesaconase (EC: 4.2.1.34)
52a, 52c (Figure 8) and 19a (Figure 9)	K00027 malate dehydrogenase (oxaloacetate-decarboxylating) [EC: 1.1.1.38]
52 b	K01571 oxaloacetate decarboxylase (Na+ extruding) subunit alpha [EC: 7.2.4.2]
52 b	K01003 oxaloacetate decarboxylase [EC: 4.1.1.112]
52c (Figure 8) and 19 (Figure 9)	K00028 malate dehydrogenase (decarboxylating) [EC: 1.1.1.39]

Footnote: *—reactions 67–68 included in Table 5.

**Table 7:** Pathway of propionyl-CoA formation from acetyl-CoA: the enzymes potentially involved in the synthesis of propionyl-CoA through the some reactions common for 3-hydroxypropionate and 3-hydroxypropionate/4-hydroxybutyrate cycles.

Reaction Number in Figure 8	Function Number, Enzyme Name and Number (KEGG Orthology Database)
69	K02160 acetyl-CoA carboxylase biotin carboxyl carrier protein
	K01961 acetyl-CoA carboxylase, biotin carboxylase subunit [EC: 6.4.1.2 6.3.4.14]
	K01962 acetyl-CoA carboxylase carboxyl transferase subunit alpha [EC: 6.4.1.2 2.1.3.15]
	K01963 acetyl-CoA carboxylase carboxyl transferase subunit beta [EC: 6.4.1.2 2.1.3.15]
70	K14468 malonyl-CoA reductase/3-hydroxypropionate dehydrogenase (NADP+) [EC: 1.2.1.75 1.1.1.298]
70	K14468/K15017 malonyl-CoA reductase (malonate semialdehyde-forming) [EC: 1.2.1.75 1.1.1.298]
70a	No function number in KEGG; malonate CoA-transferase [EC: 2.8.3.3]
70a	K20511 malonyl-S-ACP:biotin-protein carboxyltransferase subunit MadD [EC: 2.1.3.10]
	K20510 malonyl-S-ACP:biotin-protein carboxyltransferase subunit MadC [EC: 2.1.3.10]
	K13931 malonate decarboxylase delta subunit
	K13929 malonate decarboxylase alpha subunit [EC: 2.3.1.187]
70a	K01578 malonyl-CoA decarboxylase [EC: 4.1.1.9]
70b	No function number in KEGG;malonate-semialdehyde dehydrogenase [EC: 1.2.1.15]
71	No function number in KEGG; 3-hydroxypropionate dehydrogenase [EC: 1.1.1.59]
71	K18602 malonic semialdehyde reductase [EC: 1.1.1.-]
72, 73, 74	K14469 acrylyl-CoA reductase (NADPH)/3-hydroxypropionyl-CoA dehydratase/3-hydroxypropionyl-CoA synthetase [EC: 1.3.1.84 4.2.1.116 6.2.1.36]
72	K18594 3-hydroxypropionyl-CoA synthetase (ADP-forming) [EC: 6.2.1.-]
72	K05605 3-hydroxyisobutyryl-CoA hydrolase [EC: 3.1.2.4]
73	K01782 3-hydroxyacyl-CoA dehydrogenase/enoyl-CoA hydratase/3-hydroxybutyryl-CoA epimerase [EC: 1.1.1.35 4.2.1.17 5.1.2.3]
73	No function number in KEGG; fatty acid oxidation complex, α-subunit [EC: 1.1.1.35; 4.2.1.17; 5.3.3.8]
73	K01715 enoyl-CoA hydratase [EC: 4.2.1.17]
74	K00249 acyl-CoA dehydrogenase [EC: 1.3.8.7]
74	K19745 acrylyl-CoA reductase (NADPH) [EC: 1.3.1.-]

**Table 8:** Glyoxylate to malate converting enzymes (reactions 1 or reaction 2–3); PEP formation from glyoxylate in the glycerate pathway (reactions 32–35); OAA formation from glyoxylate in the β-hydroxyaspartate cycle (reaction 28–31, 28).

Reaction Number in Figure 9	Function Number, Enzyme Name and Number (KEGG Orthology Database)
1	K01638 malate synthase [EC: 2.3.3.9]
2 (Figure 9) and 68,53 (Figure 8)	K08691 malyl-CoA/(*S*)-citramalyl-CoA lyase [EC: 4.1.3.24 4.1.3.25] *
3 (Figure 9)	K14451 (3*S*)-malyl-CoA thioesterase [EC: 3.1.2.30] *
28	No function number in KEGG; aspartate-glyoxylate aminotransferase [EC: 2.6.1.35]
29	K18425 β-hydroxyaspartate aldolase [EC: 4.1.3.41]
30	No function number in KEGG; β-hydroxyaspartate dehydratase
31	No function number in KEGG; iminosuccinate reductase (EC: 1.4.1.-)
32	K01608 tartronate-semialdehyde synthase [EC: 4.1.1.47]
33	K00042 2-hydroxy-3-oxopropionate reductase [EC: 1.1.1.60]
34	K00865, K11529 glycerate 2-kinase [EC: 2.7.1.165]
35	K01689 enolase [EC: 4.2.1.11]

Footnote: *—Designations—see note to Table 5.

**Table 9:** PA and PEP carboxylating enzymes (reaction 19, 19a, 20–22); pathway of fumarate (reactions 23–25) or (*S*)-malate (reactions 23, 26) formation from PA; pathway of *cis*-aconitate formation from PA and acetyl-CoA (reactions 15–17, 18 (or 18a, 18b)).

Reaction Number in Figure 9	Function Number, Enzyme Name and Number (KEGG Orthology Database)
19a, 19 and (Figure 9) 52a, 52c (Figure 8)	K00027 malate dehydrogenase (oxaloacetate-decarboxylating) [EC: 1.1.1.38]
19a, 19	K00029 malate dehydrogenase (oxaloacetate-decarboxylating) (NADP+) [EC: 1.1.1.40]
19 (Figure 9) and 52c (Figure 8)	K00028 malate dehydrogenase (decarboxylating) [EC: 1.1.1.39]
20	K01610 phosphoenolpyruvate carboxykinase (ATP) [EC: 4.1.1.49]
20	K01596 phosphoenolpyruvate carboxykinase (GTP) [EC: 4.1.1.32]
20	K20370 phosphoenolpyruvate carboxykinase (diphosphate) [EC: 4.1.1.38]
21	K01595 phosphoenolpyruvate carboxylase [EC: 4.1.1.31]
22	K01958 pyruvate carboxylase [EC: 6.4.1.1]
23	K07246 tartrate dehydrogenase/decarboxylase/d-malate dehydrogenase [EC: 1.1.1.93 4.1.1.73 1.1.1.83]
24	No function number in KEGG; maleate hydratase EC 4.2.1.31
25	K01799 maleate isomerase [EC: 5.2.1.1]
26	No function number in KEGG; (*R*)/(*S*)-malate racemase
15, 2 (Figure 9) and 68, 53 (Figure 8)	K08691 malyl-CoA/(*S*)-citramalyl-CoA lyase [EC: 4.1.3.24 4.1.3.25] *
16	K18290 itaconyl-CoA hydratase [EC: 4.2.1.56]
17	K01902 succinyl-CoA synthetase alpha subunit [EC: 6.2.1.5]
17	No function number in KEGG; succinyl-CoA:itaconate CoA-transferase (EC: 2.8.3.-)
18	K17724 aconitate decarboxylase [EC: 4.1.1.6]

Footnote: *—Designations—see note to Table 5.

**Table 10:** Pathways of propionyl-CoA conversion into succinyl-CoA or PA: methylmalonyl-CoA pathway (reactions 4–6); pathway of propionyl-CoA oxidation via lactate to PA (reaction 7–10); methylcitrate cycle (reactions 11, 12 (or 12a-12b), 13, 14).

Reaction Number in Figure 9	Function Number, Enzyme Name and Number (KEGG Orthology Database)
4	K01965 propionyl-CoA carboxylase alpha chain [EC: 6.4.1.3]
	K01966 propionyl-CoA carboxylase beta chain [EC: 6.4.1.3 2.1.3.15]
4	K03416; K17489; K17490 methylmalonyl-CoA carboxytransferase [EC: 2.1.3.1]
5	K05606 methylmalonyl-CoA/ethylmalonyl-CoA epimerase [EC: 5.1.99.1]
6	K01847 methylmalonyl-CoA mutase [EC: 5.4.99.2]
7	K14469, K15020 acrylyl-CoA reductases (NADPH) [EC: 1.3.1.84]
7	K01039, K01040 glutaconate CoA-transferases (EC: 2.8.3.12)
7	K20143 acrylyl-CoA reductase (NADH) [EC: 1.3.1.95]
7	No function number in KEGG; acyl-CoA-dehydrogenase
8	lactoyl-CoA- dehydratase (EC: 4.2.1.54)
9	propionate CoA-transferase (EC: 2.8.3.1)
10	K00016 L-lactate dehydrogenase [EC: 1.1.1.27]
10	K00101 L-lactate dehydrogenase (cytochrome) [EC: 1.1.2.3]
10	No function number in KEGG; malate-lactate transhydrogenase (EC: 1.1.99.7)
11	K01659 2-methylcitrate synthase [EC:2.3.3.5]
12	K01720 2-methylcitrate dehydratase [EC:4.2.1.79]
12a	K20455 2-methylcitrate dehydratase (2-methyl-trans-aconitate forming) [EC:4.2.1.117]
12b	No function number in KEGG; 2-methylaconitate isomerase (EC: 5.2.1.-)
13 (2, 3 in Figure 8)	K01681 aconitate hydratase [EC:4.2.1.3]
13	K01682 aconitate hydratase 2/2-methylisocitrate dehydratase [EC: 4.2.1.3/EC: 4.2.1.99]
14	K03417 methylisocitrate lyase [EC:4.1.3.30]

## Data Availability

Not applicable.

## Abbreviations

OAA	oxaloacetic acid
CBB cycle	Calvin–Benson–Bassham cycle
ICL	isocitrate lyase
ILV synthesis pathway	isoleucine and valine synthesis pathway
PA	pyruvate
PNSB	purple non-sulfur bacteria
Rubisco	ribulose bisphosphate carboxylase/oxygenase
rTCA cycle	reductive citric acid cycle
TCA cycle	tricarboxylic acid cycle (or the citric acid cycle)
PGA	phosphoglycerate
PFL	pyruvate formate lyase
